# Metaproteomics reveal that rapid perturbations in organic matter prioritize functional restructuring over taxonomy in western Arctic Ocean microbiomes

**DOI:** 10.1038/s41396-019-0503-z

**Published:** 2019-09-06

**Authors:** Molly P. Mikan, H. Rodger Harvey, Emma Timmins-Schiffman, Michael Riffle, Damon H. May, Ian Salter, William S. Noble, Brook L. Nunn

**Affiliations:** 1 Ocean, Earth and Atmospheric Sciences, Old Dominion University, 406 Oceanography & Physical Sciences Building, Norfolk, VA 23529 USA; 2 Department of Genome Sciences, University of Washington, William H. Foege Hall, 3720 15th Ave NE, Seattle, WA 98195 USA; 3 Department of Biochemistry, University of Washington, 1705 NE Pacific St., Seattle, WA USA; 4 Faroese Marine Research Institute, Nóatún 1, FO-100 Tórshavn, Faroe Islands; 5 Alfred Wegener Institute Helmholtz Center for Polar and Marine Research, Bremerhaven, Germany

**Keywords:** Microbial ecology, Proteomics, Microbial biooceanography, Marine microbiology

## Abstract

We examined metaproteome profiles from two Arctic microbiomes during 10-day shipboard incubations to directly track early functional and taxonomic responses to a simulated algal bloom and an oligotrophic control. Using a novel peptide-based enrichment analysis, significant changes (*p*-value < 0.01) in biological and molecular functions associated with carbon and nitrogen recycling were observed. Within the first day under both organic matter conditions, Bering Strait surface microbiomes increased protein synthesis, carbohydrate degradation, and cellular redox processes while decreasing C1 metabolism. Taxonomic assignments revealed that the core microbiome collectively responded to algal substrates by assimilating carbon before select taxa utilize and metabolize nitrogen intracellularly. Incubations of Chukchi Sea bottom water microbiomes showed similar, but delayed functional responses to identical treatments. Although 24 functional terms were shared between experimental treatments, the timing, and degree of the remaining responses were highly variable, showing that organic matter perturbation directs community functionality prior to alterations to the taxonomic distribution at the microbiome class level. The dynamic responses of these two oceanic microbial communities have important implications for timing and magnitude of responses to organic perturbations within the Arctic Ocean and how community-level functions may forecast biogeochemical gradients in oceans.

## Introduction

In the surface ocean, primary production driven by phytoplankton growth dynamics is the essential process for the transfer of carbon from inorganic to organic pools and structures the food web for higher trophic consumers. While a fraction of this organic material (OM) supports upper trophic levels, the microbial loop recycles the majority of OM in the water column [1]. Linking microbial functionality to essential biogeochemical cycles has remained a primary objective of microbial ecology for decades. This functionality is predominantly regulated by a complex mixture of bacteria, archaea, and eukarya. In particular, differential responses of bacteria to organic substrates have led to the observation that the heterotrophic community and associated core metabolic genes may be structured by organic substrate availability [2]. As the complexity and often trace-level concentrations of thousands of potential substrates make them a challenge to track in the ocean, researchers are exploring the use of technologies to track the physiological response of microbial communities to changing chemical compositions in the local environment as well as the dynamic relationship between microbiota and the surrounding ecosystem [2–5].

Since proteins carry out the majority of molecular functions and are tightly regulated within the cell, their characterization, quantification, and timing of expression can serve as a biologically relevant proxy for the organism’s current phenotype. Several studies have successfully linked bacterial metaproteomic (i.e., community proteomic) responses to important biogeochemical cycles in situ, reflecting temporally relevant metabolic strategies of natural microbiomes to their environment, with some reporting high taxonomic resolution [5–10]. Nevertheless, as most metaproteomic analysis pipelines are adaptations of traditional single-species proteomic approaches, there are inherent complications that emerge when multiple species are analyzed in a single sample [11, 12], in particular, the assignment of an identified peptide to multiple protein sequences from the provided genome [13, 14]. In the case of a native oceanic microbiome where many species are present and few are cultured, a single peptide can be conserved across many proteins which may differ in predicted functions and even map to different proteins across multiple species, genera, families, or even phyla [15–19].

Here we report the response of two Arctic microbial communities to rapid changes in organic availability typical of polar environments using a novel metaproteomic approach over 10-day shipboard incubations. Before experimental manipulations were initiated, metagenomes of the native microbial population were completed to generate a site-specific reference database for peptide identification followed by mass spectrometry-based metaproteomics on incubation samples to track temporal functional responses through time [17]. Specifically, the new methodology used resolves the metaproteomics technical challenge of protein inference and, importantly, allows a discovery-based peptide-centric approach [20] to address the critical need to identify relevant metabolic strategies and identify significantly changing functions in complex microbiomes. Once those changing functions were revealed, taxonomy was assigned using the peptide data and was supported by 16S rRNA gene sequencing. With this methodology, the accuracy in reporting functions distributed among different taxonomic groups of a mixed community is increased, the statistical robustness is enhanced, and the resolution is more amenable to large-scale functional-modeling efforts.

The simultaneous measurement of taxonomic and functional shifts without limiting the analysis to specific taxonomic groups or processes allowed the comprehensive metabolic response of the native Arctic microbial community to be determined over time. With this novel method, we demonstrate that complex marine microbiomes collected from the shallow shelf system of the western Arctic Ocean undergo rapid functional restructuring related to carbon (C) and nitrogen (N) cycling after perturbations to their organic substrate environments which reveal implications for broader biogeochemical cycles.

## Methods

### Seawater sample collection and shipboard incubations with organic amendments

Seawater was collected from the subsurface chlorophyll maximum (SCM) of the Bering Strait and the bottom waters of the Chukchi Sea (Fig. S1) as described in May et al. [21] and detailed in SI. Briefly, water was collected from surface and bottom waters at sites with unique physicochemical parameters to target taxonomically distinct microbiomes (Fig. S2). These were filtered sequentially through 10.0 and 1.0 µm filters to isolate free-living bacteria from large eukaryotic grazers and remove particulate organic matter. At each site 1.0 µm prefiltered seawater was subsequently incubated shipboard for 10 days at 0 °C in the dark with 40 L of seawater from each location distributed in two, 20 L carboys as duplicate treatments. One treatment received no additions while the other was supplemented with in situ algal organic matter (Table S1), collected and concentrated from the Bering Strait site SCM (5.0–10.0 µm) then frozen to lyse cells. As it included both particulate and dissolved fractions of the lysed cells, this experimental treatment is referred to as “OM input”. The nonamended treatment of 20 L of from each location (also 1.0 µm filtered) served as a control to examine bacterial responses to incubation conditions and residual dissolved substrates. Subsamples for metaproteomics (0.2 µm polycarbonate (PC) filters—Whatman Nuclepore) were collected from the in situ (initial) microbiomes and on days 1, 6, and 10. Samples for 16S rRNA gene sequencing (0.22 μm Sterivex cartridges—Millipore) were collected on days 0, 1, 2, 4, 6, and 10. Further details plus bacterial abundance and compound analysis methodologies can be found in SI.

### 16S rRNA gene sequencing, DNA library construction, sequencing, and bioinformatics

Methods for 16S rRNA gene isolation, amplicon sequencing, and bioinformatics followed Fadeev et al. [22] and are detailed in SI. Briefly, samples of bacterial DNA were isolated from filters prior to PCR amplification (MO BIO Laboratories, Inc., Carlsbad, CA, USA). Library preparation was performed according to Illumina: 16S Metagenomic Sequencing Library Preparation instructions (Illumina, Inc., San Diego, CA, USA). 16S rRNA sequences were obtained on the Illumina MiSeq in a 2 × 300 bp paired-end run and in a 2 × 250 bp paired-end run on the Illumina HiSeq (CeBiTec Bielefeld, Germany). After quality trimming and filtering [23, 24], clustering into OTUs was completed [25] and one representative sequence per OTU was taxonomically classified at a minimum alignment similarity of 0.9, and a last common ancestor consensus of 0.7 [26]. Nonbacterial OTUs and those with a single sequence were excluded. Raw paired-end sequence, primer-trimmed reads are in the European Nucleotide Archive (ENA; https://www.ebi.ac.uk/ena) [27] under the project accession number PRJEB33210.

### Metagenomics: sample preparation and data analysis

To produce a protein sequence database to which all tandem mass peptide spectra were correlated, a microbial metagenome was completed by combining 7 L of filtered seawater from both the Bering Strait and Chukchi Sea (0.2 µm PC filters). Methodological details are outlined in SI. Briefly, DNA was extracted following the protocol in Wright et al. [27], library preparation was completed using the Kapa Hyper Kit [21] and sequenced on an Illumina HiSeq 2500 (PE100) in one lane. Raw sequencing reads can be found in NCBI’s Short Read Archive: SRP071900. MOCAT was used to process, assemble and translate raw reads, and generate protein sequences [28]. The metagenome-predicted protein database is available at ProteomeXchange Consortium via the PRIDE [29] partner repository (https://www.ebi.ac.uk/pride/archive/projects/PXD008780).

### Metaproteomics: sample preparation and data analysis

Metaproteomic sample preparation and liquid chromatography and tandem mass spectrometry (LC-MS/MS) are outlined in Timmins-Schiffman et al. [17] and SI. Briefly, filters were submerged in 100 µl of 6 M urea and 600 µl of 50 mM NH_4_HCO_3_ and sonicated (5 × 20 s) to lyse cells. Proteins within the lysate were reduced and alkylated using dithiothreitol and iodoacetamide, respectively, digested with Trypsin (12 h; 1:20 enzyme to protein) and desalted with C18 centrifugal spin columns. Peptides were resuspended in 2% ACN, 0.1% formic acid prior to analysis with a nanoAcquity UPLC (Waters Corp., Milford, MA) in line with a Q-Exactive-HF (Thermo Fisher Scientific, Waltham, MA). The mass spectrometry data is available through ProteomeXchange (PXD008780). All database searches were performed using Comet [30] against the sample-specific Bering Strait/Chukchi Sea metagenome-derived proteome database [17]. Peptide spectrum matches (PSMs) were retained at a 1% false discovery rate with the Percolator algorithm [31, 32].

### Peptide-based Gene Ontology (GO) enrichment analysis

The abundance of GO functional categories [33, 34] was quantified using the methods described by Riffle et al. [20] and outlined in SI. Briefly, each peptide was associated with all metagenome proteins containing it, and GO annotations of each top match (and their ancestors) were used to construct a directed acyclic graph (DAG) containing all GO terms associated with the peptide, and the spectral count for each GO term was increased by the spectral count of the peptide.

To determine the relative contribution of each taxon to each GO term, every peptide was assigned a lowest common ancestor (LCA) of each top BLAST hit for the metagenome proteins containing the peptide (open software 2018: MetaGOmics [20]). The spectral counts for the LCA and all ancestor taxa were incremented by the spectral count for each respective peptide, and this spectral count was divided by the spectral count for the GO term to produce a proportion of all spectra for a GO annotation that was unambiguously contributed by each taxon. Although tables with all temporal taxonomic distributions for functions are provided (Supplemental Datasets 1–4), we report functional changes at the class level, which encompasses 85% of the peptide evidence; reporting data at the genus or family level would have resulted in a 53 or 33% loss in total reportable peptide data, respectively (Fig. S3). When peptides could not be matched to a taxon or were matched to an LCA less granular than class (e.g., phylum), the difference was assigned to an Unclassified taxonomic group. Nonbacterial PSMs were removed from further analysis (Table S2). The rate of change of taxonomic classes based on peptide data was determined using a matrix of the PSM data where each row represented a class and time points were column headers. The cells in the matrix comprised the ratio of PSMs in the given time point that could be unambiguously assigned to that class. Average rate of change was calculated as the sum of the absolute values of the difference in the ratios from time points 0 to 1 and 1 to 6 divided by the number of days between time points (i.e., |(day_6_−day_1_)|/5_days_). The mean of the rates of change was then calculated for the three different GO aspects (e.g., biological process, cellular component, and molecular functions) and are reported. The calculations were repeated for the 71 reported GO terms to allow us to compare the taxonomic rate of change with the functional rate of change (Table S3, Dataset 5). To determine if the taxonomic rate of change was significantly different from the functional rate of change within each incubation, the matrices of functional PSM ratios were permuted (at the row level) 10,000 times to empirically estimate a null distribution and used to calculate a *p*-value for observing the observed difference in rates (python script with Dataset 5).

Enrichment analysis of GO functions was performed using metaGOmics [20] and outlined in SI. Briefly, the log_2_-fold change of Laplace-corrected GO term spectral counts were compared between each pair of mass spectrometry runs. For this study, we compared sequential time points within each experiment (i.e., initial Bering Strait sample vs. day 1, day 1 vs. day 6, and day 6 vs. day 10). Terminal GO terms (those most specific in the DAG) with Bonferroni-corrected *p*-value < 0.01 from a two-tailed test of proportions were considered significant and included in the enrichment analysis.

## Results

### Peptide and 16S rRNA taxonomic assignments

**Fig. 1:**
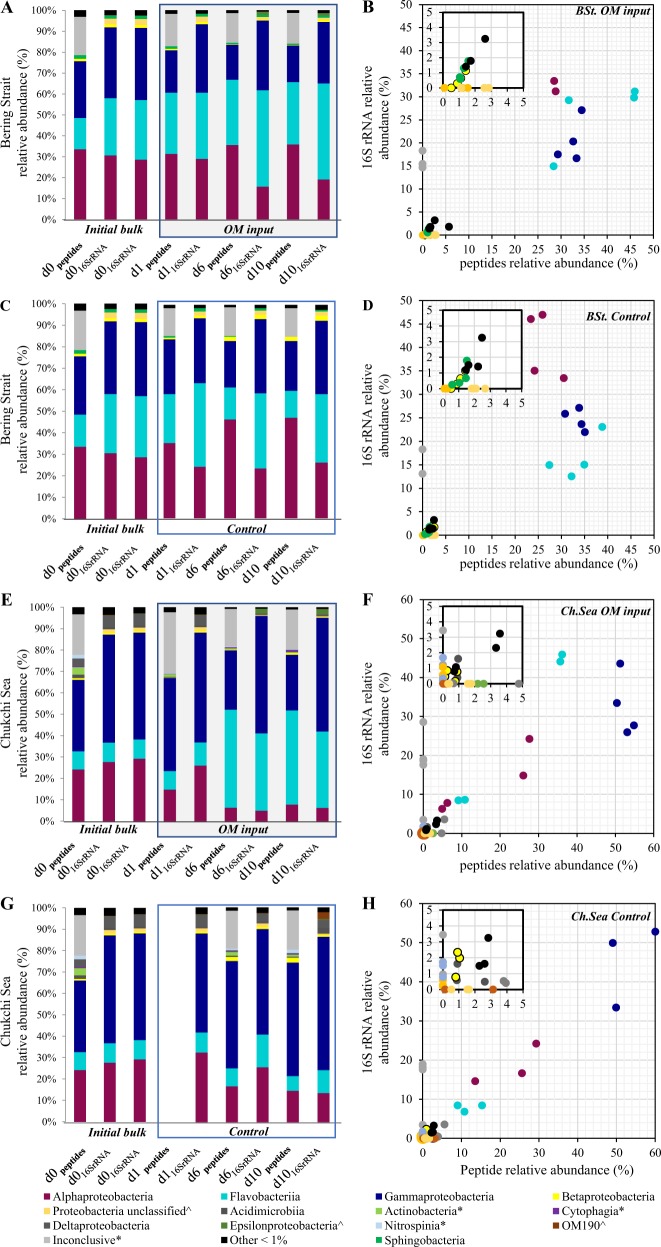
Taxonomic distributions in Bering Strait (**a–d**) and Chukchi Sea (**e–h**) bacterial classes under differing organic matter conditions (OM input: **a, b, e, f**; control: **c, d, g, h**) during 10 day shipboard experiments. **a, c, e, g** Relative abundance contribution of major taxonomic classes (>1%) from the proteome datasets (protein) and by 16S rRNA gene sequencing (16S rRNA). The asterisk symbol denotes classes that comprise > 1% of proteome dataset but <1% of 16S rRNA dataset; The hat symbol denotes classes that comprise > 1% of 16S rRNA dataset but <1% of proteome dataset. Control samples from the Chukchi Sea incubations at day 1 were compromised and excluded from the analysis. **b, d, f, h** Percent of peptides attributed to the major taxonomic classes compared with the percent of 16S rRNA genes identified per taxonomic class

Within the Bering Strait and Chukchi Sea microbiomes, metaproteomics data identified peptides correlating to 30 and 25 bacterial classes, respectively (Table S4) and 16S rRNA OTUs that corresponded to 53 and 63 classes, respectively (Table S5). Alphaproteobacteria, Flavobacteriia (referred to as Flavobacteria), and Gammaproteobacteria classes represented >75% of the metaproteomic identifications in the Bering Strait and 66% (Fig. 1a, c) in the Chukchi Sea incubations (Fig. 1e, g). Traditional 16S rRNA identifications showed these three classes also had similarly high contributions at over 91 and 86% of abundances, respectively. A direct comparison of the distribution of taxonomic classes identified by the peptide-based metaproteomic approach and 16S rRNA gene sequencing shows a linear Pearson’s correlation (Fig. 1b, d, f, h; BSt OM input *r*  = 0.76, *p* < 0.00001, *n* = 32; BSt Control *r* = 0.81*, p* < 0.00001, *n* *=* 32; Ch. Sea OM input *r* = 0.82*, p* < 0.00001, *n* = 56*;* Ch. Sea Control *r* = 0.76, *p* < 0.00001, *n* = *56*).

Eleven genera dominated bacterial abundances in both the Bering Strait and Chukchi Sea incubations, based on 16S rRNA OTUs across six time points (Fig. S4; Dataset 6). At the genus level, 16S rRNA gene sequencing revealed less compositional stability than at the class level and showed that community restructuring was dependent on the native initial microbiome, OM perturbation, and time. *Polaribacter* spp. increased after the addition of OM in both the Bering Strait and Chukchi Sea incubations with a temporal delay of 2 days in the Chukchi Sea and displayed inverse relative abundances with *Pelagibacter* spp. (SAR11) (*r* = −0.98, *p* < 0.01, *n* = 6), *Oceanospirillales* spp. (*r* = −0.89, p < 0.01, *n* = 6) and “Other” genera that contributed <5% abundances (*r* = −0.72, p < 0.01, *n* = 6). *Polaribacter* spp. became the dominant genus in both communities, outcompeting both abundant and less abundant genera when labile substrates were available (Fig. S4). “Other” less abundant genera composed a larger fraction of the total in the control incubations.

### Peptide-based community functions through time

**Fig. 2:**
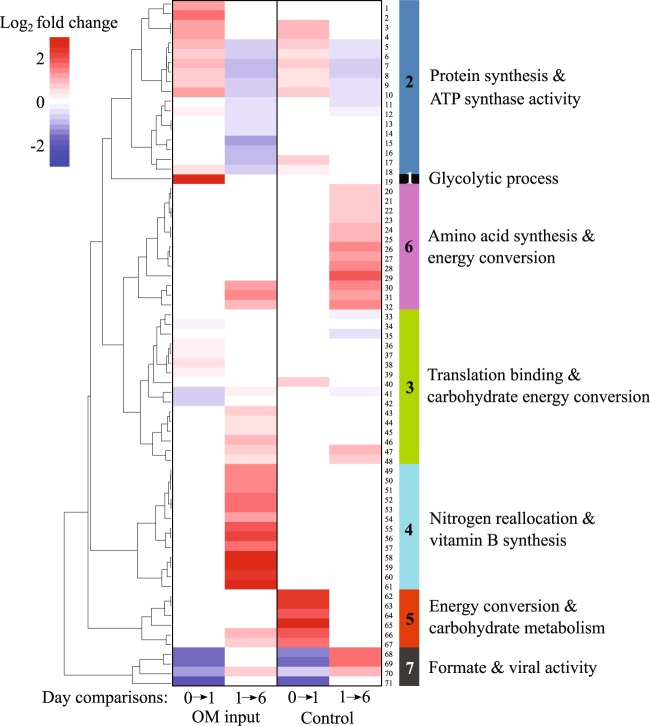
Heatmap of Bering Strait (BSt) Gene Ontology (GO) functions with significant peptide spectrum match (PSM) log_2_ fold changes (Bonferroni-corrected *p*-value < 0.01 from a two-tailed test of proportions) between time points per experiment. Column 1: initial BSt microbiome sample (indicated here as day 0) compared with day 1 with algal OM input, column 2: day 1–day 6 with OM, column 3: initial BSt (day 0) to day 1 in the control, column 4: day 1–day 6 in the control (>1.0 µm removed). Color shading indicates the degree of log_2_ fold change as seen in the color key. Colored bars on the left indicate the seven clusters of changing functions as outlined in Table 1

Although microbial community responses can be influenced by incubation conditions (e.g., removal of grazers or container artifacts [35]), only significantly changing functions identified through comparing site-matched incubations were analyzed to minimize reporting of artifact-associated functions. Within the Bering Strait and Chukchi Sea microbiomes, tens of thousands of PSMs matched to thousands of GO functions (Table S6), which were identified at high functional and taxonomic resolution (Datasets 1–4). To identify significantly changing functions through time in an unbiased manner, GO functional assignments and class-level taxonomic information were extracted. Focusing on class-level data allowed the use of the greatest percentage of the peptide results (Fig. S3) and provided a consistent framework to compare the temporal progression of functions and metabolically active bacteria. In the Bering Strait microbiome, the peptide-based enrichment analysis of terminal GO terms between time points identified 71 functions with significant changes in abundance (*p*-value < 0.01); these functions generated seven hierarchical clusters that exhibit time-dependent functional processes linked to carbon and nitrogen cycling after OM perturbations (Fig. 2; Table 1, S7). The Bering Strait community functional and compositional responses primarily occurred within the first 6 days after perturbation and were largely maintained to the end of the incubations.

**Table 1:** Gene Ontology (GO) functions that changed significantly over time (Bonferroni-corrected *p*-value < 0.01 from a two-tailed test of proportions) within the Bering Strait (BSt) incubations

Substantial community-wide proteome remodeling was observed under both the simulated algae bloom (OM input) and the oligotrophic (control: OM was removed) treatments in the Bering Strait (Fig. 2). The addition of OM resulted in 54 significantly changing bacterial functions over the incubation period while only 41 changed in the control (Fig. 2). Of these significantly changing GO terms, 24 were identified in both the OM input and control incubations. However, the timing and degree of these 24 responses were highly variable as the incubation proceeded. The mean rate of change between the ratios of peptide spectral matches for given GO terms expressed per day by the Bering Strait microbiome with OM input was 0.0235 (*n* = 71), while the average rate of change per day in the control was 0.0143 (*n* = 71) (Table S3). Functional change occurred more rapidly than taxonomic change (*p* < 0.0001) with the rate of change of peptides at the taxonomic level for the 24 classes at 0.008 (*n* = 6), <35% of the rate of change of peptides for the 71 functions (Table S3).

In both Bering Strait OM addition and control incubations on day 1, there was an increase in 10 protein synthesis-related GO functions (functions #3–12, cluster 2) (Fig. 2; Table 1). A simultaneous increase in ten carbohydrate metabolism functions (functions #19, #36–39, #62–67) also occurred, with some of the largest changes within the first day (log_2_ fold of 2.2–3.8; Fig. 2; Table 1, S7). The increase in glycolysis-related peptides (function #19) and corresponding essential functions (#36–39, cluster 3) observed after the algal OM input to provide cells with adenosine triphosphate (ATP) [36, 37]. Similarly, in the day 1 control a cluster of functions involved in the electron transport chain for energy flow and storage (#62–65, cluster 5) increased with carbohydrate metabolism (functions #66–67), while C1 metabolism peptides decreased: NAD^+^-formate dehydrogenase (FDH) and molybdenum (Mo) ion binding (#68–69, cluster 7).

By day 6 in both the Bering Strait incubation treatments, protein synthesis (cluster 2) declined while increases were observed in metabolic functions related to energy production and resource utilization, including the ATP-binding cassette (ABC) transporter complex (function #31), TCA cycle (functions #43–48, cluster 3), and formate C1 catabolism (functions #55–57 under OM input; #68–69 within the control). Although similar protein and ATP synthesis functional responses were observed in the Bering Strait OM input and the control, Day 6 also saw divergent nitrogen regulation and uptake metabolisms (Fig. 2). OM input incubations stimulated log_2_-fold changes ≥2 in nitrogen regulation and transport (Fig. 2 cluster 4: #49, #51, #53–54, #58–60) and vitamin-B synthesis (cluster 4: #50, #55–57, #61). Specifically, the synthesis of thiamine (vitamin-B1), a crucial vitamin and coenzyme involved in essential metabolic processes including amino acid and carbon metabolism and the regulation of gene expression [38], increased approximately fourfold (function #61). In addition, by day 6 after OM input, cluster 4 exhibited increases in peptides correlated to pyridoxal phosphate (vitamin-B6) binding (function #50), formate tetrahydrofolate (THF; functions #55-57) (involving vitamin-B9), glutamine synthetase (GS) (functions #49, #51), glutamate synthase (glutamine:2-oxoglutarate aminotransferase, GOGAT) (functions #58, #60), and N-fixation (function #54). Control incubations at day 6 simultaneously increased mobilization of amino acids (cluster 6) through catabolism and transport pathways, and C1-based energy production through formate oxidation (FDH/Mo) (functions #68–69, cluster 7). In summary, the absence of OM input in Bering Strait control exhibited less proteome remodeling, a decreased rate of functional change and minor taxonomic changes in bacterial genera over time.

### Taxonomy responsible for significantly changing functions

**Fig. 3:**
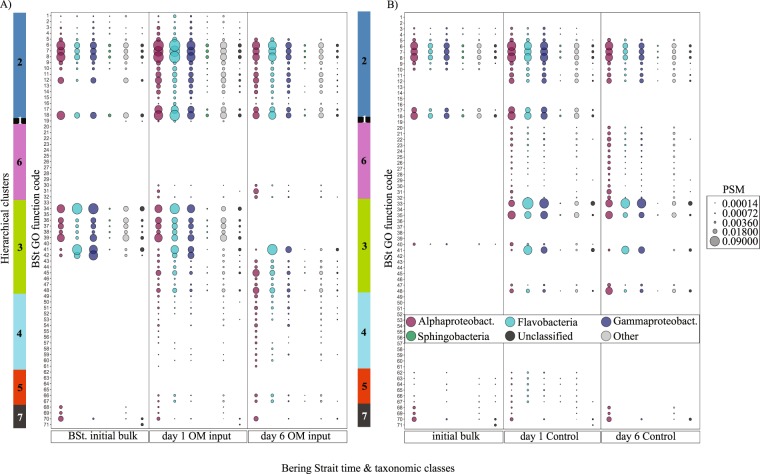
Peptide spectrum match (PSM) values for the six major Bering Strait (BSt) microbiome taxonomic categories showing greatest PSM counts associated with the 71 significantly changing GO functions as defined by Bonferroni-corrected *p*-value < 0.01 from a two-tailed test of proportions. **a** Shipboard incubations with algal particulate organic matter input (OM) and **b** the control incubation. Sizes of bubbles are scaled to PSM counts by area. Missing data for all classes for a given time point (e.g., 13–17 for initial BSt sample, BSt) indicates that no significant changes were measured between that time point and the next time point (e.g., day 1 OM). Alphaproteobact. = Alphaproteobacteria; Gammaproteobact. = Gammaproteobacteria. Clusters with function code identifiers are presented in Table 1. All PSM data to accompany this Fig. are available in Datasets 1 and 2, including a detailed breakdown of the “Other” category

**Fig. 4:**
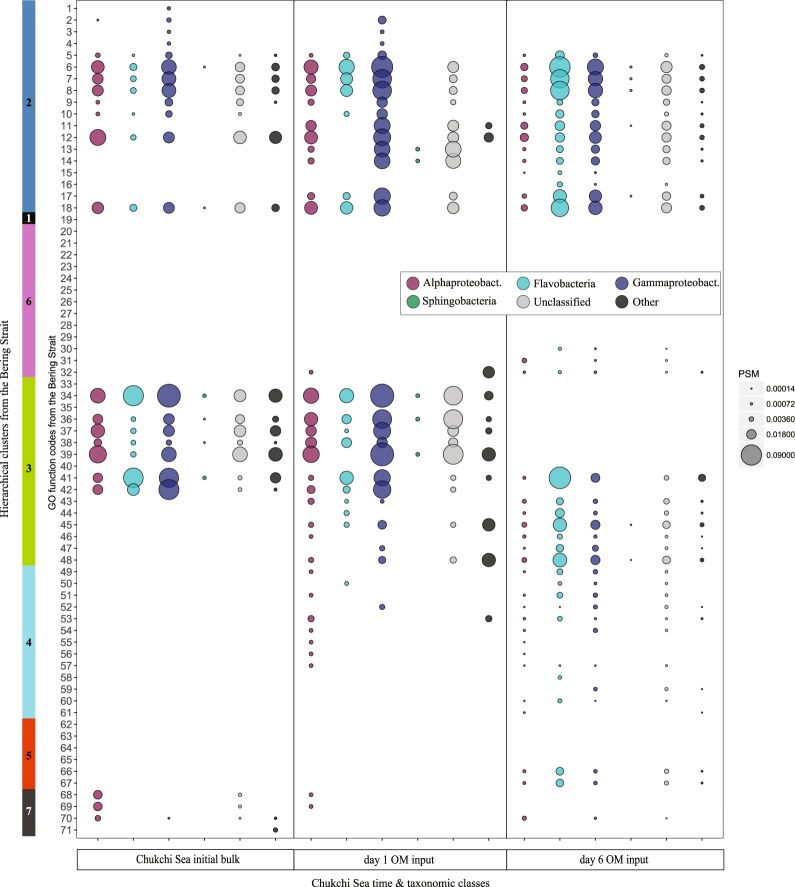
Peptide spectrum match (PSM) values for the six major Chukchi Sea microbiome taxonomic categories showing greatest PSM counts associated with the 71 significantly changing GO functions as defined by Bonferroni-corrected *p*-value < 0.01 from a two-tailed test of proportions. Shipboard incubations with algal OM input are scaled to PSM counts by area. Missing data for all classes for a given time point indicates that no significant changes were measured between that time point and the next time point. Alphaproteobact. = Alphaproteobacteria; Gammaproteobact. = Gammaproteobacteria. Clusters with function code identifiers are presented in Table 1. Additional data including PSM counts and a detailed breakdown of the “Other” category are shown in Datasets 1 and 2

Since every peptide has an associated functional and taxonomic annotation, each GO term function identified as significantly changing could be further parsed to determine the associated taxonomic classes (i.e., Figs. 3, 4). In both Bering Strait OM input and controls, the rate of change in the ratio of peptides associated with different taxonomic classes per day was 0.007–0.008; significantly less than the rate determined for functional terms (*p* < 0.0001; Table S3). In general, OM input to Bering Strait incubations rapidly increased the relative abundances of Flavobacterial peptides on day 1 (15% to >23%) where they remained ~30% of the total (Fig. 1a, b). 16S rRNA gene-sequencing data also revealed that this class was dominated by *Polaribacter* spp. genus (30%; Fig. S4). Carbohydrate metabolism and protein synthesis at day 1 increased the most from day 0 in Flavobacteria, contributing to the increased abundance of this class in Bering Strait incubations, even though Alphaproteobacteria was associated with a larger percentage of peptides for many of these functions (Fig. 3; #36–39, Dataset 1 and 2). As the incubation progressed to day 6, Alphaproteobacteria dominated the increase in ABC transport complexes under both treatments (Fig. 3; #31), and Alphaproteobacteria-assigned peptides were important drivers of the observed shift in nitrogen transport, regulation and metabolism, plus vitamin synthesis (Fig. 3 cluster 4).

### Chukchi Sea bottom water microbiome

Despite geographic separation and depth of origin in Bering Strait and Chukchi Sea communities, OM input stimulated a similar increase in bacterial cell numbers (Table S1) and the same three taxonomic classes comprised the core microbiomes (Fig. 1; Dataset 3). Nevertheless, community-wide metabolic functions were not identical (Fig. S5, Tables S7 and S8) and the Chukchi Sea was primarily controlled by Gammaproteobacteria (Fig. 1). For example, Chukchi Sea Gammaproteobacteria dominated the observed changes in peptide-based metabolic activity seen at day 1 (Fig. 4). Although many of the GO terms identified to be significantly changing in the Chukchi Sea were parent or sibling GO terms to those identified in the Bering Strait microbiome, there was a temporal delay in their increased abundance (e.g., peptides associated with multiple translation and carbohydrate metabolism terms increased at day 6 in the Chukchi Sea compared with day 1 in the Bering Strait; Fig. S5, Table S8). In response to OM input, the average functional rate of change per day of 0.0208 in the Chukchi Sea was comparable with the rate of change measured in the Bering Strait (Table S3). However, the rate of change in the ratio of peptides associated with different classes per day in the Chukchi Sea OM input incubation was over double those within the Bering Strait and not significantly different than the mean rate of change determined for the functional terms (Table S3).

## Discussion

### Initial response to OM input: carbon acquisition

Within the Bering Strait incubations, functional rates of change were calculated to be significantly higher than the taxonomic rate of change which suggests that OM perturbation directs initial community functionality before major alterations to the taxonomic distribution occur. Within the first day, functional responses show that carbohydrates were among the first diatom-derived OM substrates bioavailable to the Bering Strait microbiome as predicted for marine bacteria (e.g., [4, 39, 40]). Carbon acquisition also fueled protein synthesis by day 1; this paired first response has been previously observed in bacteria reacting to OM stimulus [2, 41–43] and is known to drive increases in ribosomal proteins that are important indicators of bacterial growth [44].

Although Flavobacteria began at relatively low abundance, they appeared to effectively compete with other bacterial taxa in both microbiomes by rapidly assimilating available carbohydrates, fueling biomass production in incubations that received algal OM (Fig. 1a, b, e, f). Flavobacteria are known to exploit complex OM with a diverse array of compound-specific enzymes and are capable of motility and substrate adhesion in the North Atlantic [45]; these results demonstrate Flavobacteria operate effectively in polar waters as well. At a higher taxonomic resolution, it was evident that *Polaribacter* spp. (of Flavobacteria) in particular benefited from the bloom simulation, as their relative 16S rRNA sequence abundances reached 30% by day 2 and they effectively outcompeted both more and less abundant genera (Fig. S4). This genus has been shown to upregulate enzymes that hydrolyze poly- and monosaccharides following phytoplankton blooms [6, 46, 47], supporting the observation here that their early increase in abundance resulted from a specialized strategy to rapidly access energy from algal-derived organic substrates. These functionalities likely enhance their access to particulate and dissolved OM, providing a competitive advantage over other community members in systems subject to episodic influxes of OM from seasonal production.

Another competitive advantage revealed in the peptide results was seen in the Alphaproteobacteria on days 1 and 6 as they dominated the increase in ABC transport complexes under both OM treatments (Fig. 3, #31). This class is composed of both ubiquitous oligotrophic and copiotrophic subgroups, making it a highly diverse bacterial class with strong niche diversification throughout nutrient extremes of the global ocean [48]. ABC transporters are abundant within Alphaproteobacterial genomes [48, 49], supplying a variety of ambient monomers to these bacteria across diverse marine settings [6, 50, 51] and allowing survival under heterogeneous conditions.

### Secondary responses to OM input: nitrogen regulation

In both Bering Strait OM input and the control, decreases in protein synthesis at day 6 aligned with increases in nitrogen regulation. Alternate metabolic pathways of nitrogen acquisition were observed in the OM input vs. control experiments and suggest that microbial communities may have unique responses to decipher complex OM availability. For example, Alphaproteobacteria-assigned peptides in the OM input incubations dominated observed shifts in cellular ammonium assimilation [52, 53] and vitamin-B synthesis. This paired restructuring may represent a nitrogen-specific postbloom response by Alphaproteobacteria in the Bering Strait since these metabolic functions were not present in the control [54]. The additional increase in N-fixation enzymes was unexpected but suggests an operative mechanism to assimilate atmospheric N_2_ to increase bioavailable nitrogen. N-fixation is energetically expensive compared with the assimilation of nitrate or ammonium [55], however, *Sulfitobacter’s* dominance in this metabolic pathway (Dataset 1) may provide this genus a unique N-acquisition strategy in the region where episodic inputs of OM are routine. The high relative abundance of *Sulfitobacter* spp. seen in Arctic waters under varied OM additions, suggest N-fixation may provide a competitive advantage for this group in western Arctic waters [56].

Alternatively, there were several lines of evidence in the Bering Strait control that suggest that the microbiome had to tightly regulate the internal use of nitrogen through protein catabolism. Under reduced OM availability, regulation of amino acid metabolic pathways would provide cells with an energetically efficient mechanism to recycle carbon and nitrogen and conserve critical cellular functions [57]. ABC transporter complexes can increase cellular OM assimilation efficiency [58], representing an important bacterial response under nutrient extremes across the global ocean [50, 51, 59] and, together with protein catabolism, demonstrates which microbial functionalities confer efficiency and which help scavenge OM in low-OM (oligotrophic) environments. Increases in formate oxidation peptides (FDH/Mo) on day 6 also support the hypothesis that cellular energy was in demand when OM was limited, as this pathway represents utilization and transfer of C1 molecules for energy production [60–62]. The various enzymes involved in these divergent pathways identified in the two Bering Strait OM extremes may represent potential targets to understand microbiome carbon acquisition in the environment.

### Delayed responses by bottom water Chukchi Sea microbiome

The parallel incubations of Chukchi Sea bottom water and Bering Strait surficial waters allowed the universality of the functional and taxonomic patterns across environmental gradients to be compared. The similarities in functional responses of these spatially distinct microbiomes (GO terms and functional rate of change) indicate a high degree of overlap across polar microbiome metabolic profiles as well as taxonomic functional redundancy within the two systems. Such metabolic flexibility that allows similar taxonomic classes to fill dynamic niches by accessing complex OM under limited substrates has been previously observed in the Arctic Ocean bacterial communities [63], but here we show functional overlap also persists when OM is abundant in this ecosystem. In addition, the distinctly delayed functional response time in the Chukchi Sea incubations reveal that despite similar functional potential (i.e., genome), the bottom water community was less acclimated to receive fresh OM. The distribution of the classes responsible for the activity within the Chukchi community changed at a faster rate compared with Bering St., nearly matching the functional rate calculated in both incubations (Table S3). This suggests that the bottom water microbial community composition changed to compensate for the lack of appropriate community functionality. Importantly, the analyses of functional and taxonomic rates of change convey that microbiomes with similar dominant taxonomic profiles may differ in the rate of functional responses to environmental conditions. Further, these findings support the argument that specific bacteria can be physiologically poised to respond to a particular stimulus (e.g., [4, 45, 64, 65]) which can initiate metabolic-specific niches and divergent ecological strategies [66]. These findings have important implications for both the timing and community response to algal organic inputs and suggest that taxonomy alone (e.g., 16S rRNA) is inadequate as a predictor of functional response.

### Redundancy in functional roles across bacterial classes

Many microbial taxa adapt to fill a particular environmental niche, yet across multiple taxa, some functional redundancy may be required to maintain the stability of a complex ecosystem (e.g., [67]). This metaproteomic approach provides a time-dependent snapshot of cellular functions within a diverse microbiome, as well as insight into which taxonomic group is dominating the identified active functions (Datasets 1–4). Tracking both simulated bloom and oligotrophic environments in these two spatially distinct microbiomes revealed that many temporally controlled functions were conserved among the major taxonomic classes, irrespective of the taxonomic restructuring that was evident at the class level. In particular, this is evident in the Bering Strait microbiome incubations where the taxonomic rate of change was significantly less than the functional rate of change, indicating the native microbial distribution was functionally poised to degrade the fresh algal OM. In addition, we note that this broad redundancy in functional roles of native microbiomes could be partially due to the level of taxonomic resolution (i.e., class level) used in the present analysis (e.g., [68, 69]). Yet Aylward et al. [2] showed that even with detailed bacterial classification (i.e., OTUs), redundancies can dominate functional responses to algal dynamics across ocean basins. Although microbiome functionality did change through time, broad functional redundancy at any one time point was seen across the different taxonomic classes in response to OM perturbations.

## Conclusions

**Fig. 5:**
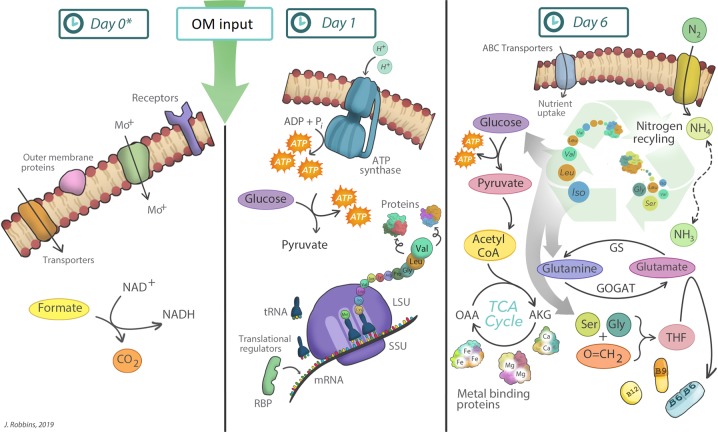
Artistic illustration of primary functions observed for the Bering Strait microbiome as a system in response to additions of algal organic matter (OM). The initial microbiome (Day 0) had a higher abundance of peptides correlating to outer membrane proteins, such as transporters and receptors, and enzymes involved in the C1 metabolic pathway. At day 0, lysed native algal organic matter was added to the incubation experiments yeilding a significant increase in peptides related to cellular growth within the microbiome observed including translation, ATP generation, and glycolysis. By day 6, significant increases in peptides associated with ABC transporters, the uptake and utilization of a range of nitrogen sources, and intracellular nitrogen recycling were identified. These pathways were inherently linked with increased abundances of peptides involved in the TCA cycle, which included increases in metal-binding proteins, GS-GOGAT pathway, and vitamin production. ADP adenosine diphosphate, ATP adenosine triphosphate, AKG alphaketogluterate, LSU large ribosomal subunit, OAA oxaloacetate, GS glutamine synthetase, GOGAT glutamine oxoglutarate aminotransferase (Glutamate synthase), SSU small ribosomal subunit, RBP ribosomal binding proteins, THF tetrahydrofolate

Understanding how the primary functions of oceanic microbiomes change spatially and temporally is incomplete without information on functional responses across broad taxonomic groups. The operative functions identified in these complex communities showed coordinated timing across bacterial classes in response to realistic algal OM input including (1) the uptake and degradation of carbon, (2) protein synthesis and ATP generation, and (3) the reallocation of cellular nitrogen and vitamin synthesis (Fig. 5). These temporal responses, many of which were observed in both Bering Strait and Chukchi Sea microbiomes soon after OM perturbations, provide an important time constraint for future field and modeling studies on organic carbon and nitrogen cycling. The recent demonstration by Coles et al. [70] that simulated microbiomes with limited functional genes can be modeled to recreate biogeochemical gradients argues for multi-“omic” data delivery. The peptide-centric method described here yielded statistically similar taxonomic distributions to 16S rRNA data at the class level, demonstrating a single technique that can access both taxonomy and active metabolism. The insights from this comprehensive enrichment approach encourage researchers to consider a complete metaproteomic dataset rather than rely on select enzymes, element-specific pathways or transporters, or taxonomic subsets of the community. The findings that many functional responses crossed major bacterial class levels at both sites suggest that functional composition, not taxonomy, may be the most relevant factor for the development of realistic biogeochemical profiles in the coastal ocean.

## Supplementary information

Table S8

Supplemental Methods and documentation

Figure S1

Figure S2

Figure S3

Figure S4

Figure S5

Table S1

Table S2

Table S3

Table S4

Table S5

Table S6

Table S7a

Table S7b

Dataset 1

Dataset 2

Dataset 3

Dataset 4

Dataset 5

Dataset 6

## References

[1] Azam F (1998). Microbial control of oceanic carbon flux: the plot thickens. Science..

[2] Aylward FO, Eppley JM, Smith JM, Chavez FP, Scholin CA, DeLong EF (2015). Microbial community transcriptional networks are conserved in three domains at ocean basin scales. Proc Natl Acad Sci USA.

[3] Konopka A, Wilkins MJ. Application of meta-transcriptomics and -proteomics to the analysis of in situ physiological state. Front Microbiol. 2012;3:1–9. 10.3389/fmicb.2012.00184 PMC3390588 22783237

[4] Poretsky RS, Sun SL, Mou XZ, Moran MA (2010). Transporter genes expressed by coastal bacterioplankton in response to dissolved organic carbon. Environ Microbiol.

[5] Bergauer K, Fernandez-Guerra A, Garcia JAL, Sprenger RR, Stepanauskas R, Pachiadaki MG (2018). Organic matter processing by microbial communities throughout the Atlantic water column as revealed by metaproteomics. Proc Natl Acad Sci USA.

[6] Teeling H, Fuchs BM, Becher D, Klockow C, Gardebrecht A, Bennke CM (2012). Substrate-controlled succession of marine bacterioplankton populations induced by a phytoplankton bloom. Science.

[7] Williams TJ, Long E, Evans F, DeMaere MZ, Lauro FM, Raftery MJ (2012). A metaproteomic assessment of winter and summer bacterioplankton from Antarctic Peninsula coastal surface waters. ISME J.

[8] Mattes TE, Nunn BL, Marshall KT, Proskurowski G, Kelley DS, Kawka OE (2013). Sulfur oxidizers dominate carbon fixation at a biogeochemical hot spot in the dark ocean. ISME J.

[9] Ng C, DeMaere MZ, Williams TJ, Lauro FM, Raftery M, Gibson JAE (2010). Metaproteogenomic analysis of a dominant green sulfur bacterium from Ace Lake, Antarctica. ISME J.

[10] Morris RM, Nunn BL, Frazar C, Goodlett DR, Ting YS, Rocap G (2010). Comparative metaproteomics reveals ocean-scale shifts in microbial nutrient utilization and energy transduction. ISME J.

[11] Mande SS, Mohammed MH, Ghosh TS (2012). Classification of metagenomic sequences: methods and challenges. Brief Bioinform.

[12] Martens L, Hermjakob H (2007). Proteomics data validation: why all must provide data. Mol Biosyst.

[13] Nesvizhskii AI, Aebersold R (2005). Interpretation of shotgun proteomic data—the protein inference problem. Mol Cell Proteom.

[14] Huang T, Wang JJ, Yu WC, He ZY (2012). Protein inference: a review. Brief Bioinform.

[15] Serang O, Moruz L, Hoopmann MR, Kall L (2012). Recognizing uncertainty increases robustness and reproducibility of mass spectrometry-based protein inferences. J Proteome Res.

[16] Tanca A, Palomba A, Deligios M, Cubeddu T, Fraumene C, Biosa G et al. Evaluating the impact of different sequence databases on metaproteome analysis: insights from a lab-assembled microbial mixture. PLoS ONE. 2013;8:1–14. 10.1371/journal.pone.0082981 PMC3857319 24349410

[17] Timmins-Schiffman E, May DH, Mikan M, Riffle M, Frazar C, Harvey HR (2017). Critical decisions in metaproteomics: achieving high confidence protein annotations in a sea of unknowns. ISME J.

[18] Saito MA, Dorsk A, Post AF, McIlvin MR, Rappe MS, DiTullio GR (2015). Needles in the blue sea: sub-species specificity in targeted protein biomarker analyses within the vast oceanic microbial metaproteome. Proteomics..

[19] Muth T, Benndorf D, Reichl U, Rapp E, Martens L (2013). Searching for a needle in a stack of needles: challenges in metaproteomics data analysis. Mol Biosyst.

[20] Riffle M, May DH, Timmins-Schiffman E, Mikan MP, Jaschob D, Noble WS et al. MetaGOmics: a web-based tool for peptide-centric functional and taxonomic analysis of metaproteomics data. Proteomes. 2018;6:1–17. 10.3390/proteomes6010002 PMC5874761 29280960

[21] May DH, Timmins-Schiffman E, Mikan MP, Haryey HR, Borenstein E, Nunn BL (2016). An alignment-free “metapeptide” strategy for metaproteomic characterization of microbiome samples using shotgun metagenomic sequencing. J Proteome Res.

[22] Fadeev E, Salter I, Schourup-Kristensen V, Nöthig E-M, Metfies K, Engel A et al. Microbial communities in the east and west fram strait during sea ice melting season. Front Mar Sci. 2018;5:1–21.

[23] Martin M (2011). Cutadapt removes adapter sequences from high-throughput sequencing reads. EMBnet J.

[24] Bolger AM, Lohse M, Usadel B (2014). Trimmomatic: a flexible trimmer for Illumina sequence data. Bioinformatics.

[25] Mahé F, Rognes T, Quince C, de Vargas C, Dunthorn M (2014). Swarm: robust and fast clustering method for amplicon-based studies. PeerJ..

[26] Pruesse E, Peplies J, Glöckner FO (2012). SINA: accurate high-throughput multiple sequence alignment of ribosomal RNA genes. Bioinformatics..

[27] Wright JJ, Lee S, Zaikova E, Walsh DA, Hallam SJ. DNA extraction from 0.22 μM Sterivex filters and cesium chloride density gradient centrifugation. J Vis Exp. 2009;31:e1352. 10.3791/1352 PMC3150049 19767717

[28] Kultima JR, Sunagawa S, Li J, Chen W, Chen H, Mende DR (2012). MOCAT: a metagenomics assembly and gene prediction toolkit. PLoS One.

[29] Vizcaino JA, Csordas A, del-Toro N, Dianes JA, Griss J, Lavidas I (2016). 2016 update of the PRIDE database and its related tools (vol 44, pg D447, 2016). Nucleic Acids Res..

[30] Eng JK, Jahan TA, Hoopmann MR (2013). Comet: an open‐source MS/MS sequence database search tool. Proteomics..

[31] Granholm V, Navarro JF, Noble WS, Käll L (2013). Determining the calibration of confidence estimation procedures for unique peptides in shotgun proteomics. J Proteom.

[32] Kall L, Canterbury JD, Weston J, Noble WS, MacCoss MJ (2007). Semi-supervised learning for peptide identification from shotgun proteomics datasets. Nat Methods.

[33] Ashburner M, Ball CA, Blake JA, Botstein D, Butler H, Cherry JM (2000). Gene Ontology: tool for the unification of biology. Nat Genet.

[34] The Gene Ontology Consortium. Expansion of the Gene Ontology knowledgebase and resources. Nucleic Acids Res. 2016;45:D331–D338. 10.1093/nar/gkw1108 PMC5210579 27899567

[35] Ferguson RL, Buckley EN, Palumbo AV (1984). Response of marine bacterioplankton to differential filtration and confinement. Appl Environ Microbiol.

[36] Castello A, Hentze MW, Preiss T (2015). Metabolic enzymes enjoying new partnerships as RNA-binding proteins. Trends Endocrinol Metab.

[37] Koebmann BJ, Westerhoff HV, Snoep JL, Nilsson D, Jensen PR (2002). The glycolytic flux in *Escherichia coli* is controlled by the demand for ATP. J Bacteriol.

[38] Jurgenson CT, Begley TP, Ealick SE (2009). The structural and biochemical foundations of thiamin biosynthesis. Annu Rev Biochem.

[39] Keil RG, Kirchman DL (1999). Utilization of dissolved protein and amino acids in the northern Sargasso Sea. Aquat Microb Ecol.

[40] Amon RMW, Fitznar HP, Benner R (2001). Linkages among the bioreactivity, chemical composition, and diagenetic state of marine dissolved organic matter. Limnol Oceano.

[41] Chin-Leo G, Kirchman DL (1990). Unbalanced growth in natural assemblages of marine bacterioplankton. Mar Ecol Prog Ser Oldendorf.

[42] Flardh K, Cohen PS, Kjelleberg S (1992). Ribosomes exist in large excess over the apparent demand for protein synthesis during carbon starvation in marine Vibrio sp. strain CCUG 15956. J Bacteriol.

[43] Muthusamy S, Lundin D, Branca RMM, Baltar F, Gonzalez JM, Lehtio J (2017). Comparative proteomics reveals signature metabolisms of exponentially growing and stationary phase marine bacteria. Environ Microbiol.

[44] Christie-Oleza JA, Fernandez B, Nogales B, Bosch R, Armengaud J (2012). Proteomic insights into the lifestyle of an environmentally relevant marine bacterium. ISME J.

[45] Gomez-Pereira PR, Schuler M, Fuchs BM, Bennke C, Teeling H, Waldmann J (2012). Genomic content of uncultured Bacteroidetes from contrasting oceanic provinces in the North Atlantic Ocean. Environ Microbiol.

[46] Pinhassi J, Azam F, Hemphala J, Long RA, Martinez J, Zweifel UL (1999). Coupling between bacterioplankton species composition, population dynamics, and organic matter degradation. Aquat Microb Ecol.

[47] Needham DM, Fuhrman JA. Pronounced daily succession of phytoplankton, archaea and bacteria following a spring bloom. Nat Microbiol. 2016;1:1–7. 10.1038/nmicrobiol.2016.5 27572439

[48] Moran MA, Belas R, Schell MA, Gonzalez JM, Sun F, Sun S (2007). Ecological genomics of marine roseobacters. Appl Environ Microbiol.

[49] Giovannoni SJ, Tripp HJ, Givan S, Podar M, Vergin KL, Baptista D (2005). Genome streamlining in a cosmopolitan oceanic bacterium. Science..

[50] Georges AA, El-Swais H, Craig SE, Li WKW, Walsh DA (2014). Metaproteomic analysis of a winter to spring succession in coastal northwest Atlantic Ocean microbial plankton. ISME J.

[51] Sowell SM, Wilhelm LJ, Norbeck AD, Lipton MS, Nicora CD, Barofsky DF (2009). Transport functions dominate the SAR11 metaproteome at low-nutrient extremes in the Sargasso Sea. ISME J.

[52] Forchhammer K (2007). Glutamine signalling in bacteria. Front Biosci.

[53] Berges JA, Mulholland MR. Enzymes and nitrogen cycling. In: Nitrogen in the marine environment. 2nd ed. Amsterdam, The Netherlands: Elsevier; 2008. p. 1385–1444.

[54] Buchan A, LeCleir GR, Gulvik CA, Gonzalez JM (2014). Master recyclers: features and functions of bacteria associated with phytoplankton blooms. Nat Rev Microbiol.

[55] Falkowski PG, Enzymology of Nitrogen Assimilation. In: Capone EJCaDG, editor. Nitrogen in the marine environment. New York, NY: Academic Press; 1983. p. 839–868.

[56] Knapp AN, Dekaezemacker J, Bonnet S, Sohm JA, Capone DG (2012). Sensitivity of Trichodesmium and Crocosphaera abundance and N2 fixation rates to varying NO3- and PO43- concentrations in batch cultures. Aquat Microb Ecol.

[57] Akashi H, Gojobori T (2002). Metabolic efficiency and amino acid composition in the proteomes of *Escherichia coli* and *Bacillus subtilis*. Proc Natl Acad Sci USA.

[58] Higgins CF (2001). ABC transporters: physiology, structure and mechanism—an overview. Res Microbiol.

[59] Sowell SM, Abraham PE, Shah M, Verberkmoes NC, Smith DP, Barofsky DF (2011). Environmental proteomics of microbial plankton in a highly productive coastal upwelling system. ISME J.

[60] Sun J, Steindler L, Thrash JC, Halsey KH, Smith DP, Carter AE et al. One carbon metabolism in SAR11 pelagic marine bacteria. PLoS ONE. 2011;6:1–12. 10.1371/journal.pone.0023973 PMC3160333 21886845

[61] Leonhartsberger S, Korsa I, Bock A (2002). The molecular biology of formate metabolism in enterobacteria. J Mol Microbiol Biotechnol.

[62] Ferry JG (1990). Formate dehydrogenase. FEMS Microbiol Lett.

[63] Alonso-Sáez L, Zeder M, Harding T, Pernthaler J, Lovejoy C, Bertilsson S et al. Winter bloom of a rare betaproteobacterium in the Arctic Ocean. Front Microbiol. 2014;5:1–9. 10.3389/fmicb.2014.00425 PMC4138443 25191307

[64] Haynes K, Hofmann TA, Smith CJ, Ball AS, Underwood GJ, Osborn AM (2007). Diatom-derived carbohydrates as factors affecting bacterial community composition in estuarine sediments. Appl Environ Microbiol.

[65] Reintjes G, Arnosti C, Fuchs BM, Amann R (2017). An alternative polysaccharide uptake mechanism of marine bacteria. ISME J.

[66] Caffrey BE, Williams TA, Jiang XW, Toft C, Hokamp K, Fares MA. Proteome-wide analysis of functional divergence in bacteria: exploring a host of ecological adaptations. PLoS ONE. 2012;7:1–12. 10.1371/journal.pone.0035659 PMC3338524 22563391

[67] Sunagawa S, Coelho LP, Chaffron S, Kultima JR, Labadie K, Salazar G et al. Structure and function of the global ocean microbiome. Science. 2015;348:1–9. 10.1126/science.1261359 25999513

[68] Fuhrman JA, Cram JA, Needham DM (2015). Marine microbial community dynamics and their ecological interpretation. Nat Rev Microbiol.

[69] Lindh MV, Figueroa D, Sjostedt J, Baltar F, Lundin D, Andersson A et al. Transplant experiments uncover Baltic Sea basin-specific responses in bacterioplankton community composition and metabolic activities. Front Microbiol. 2015;6:223–41. 10.3389/fmicb.2015.00223 PMC4381636 25883589

[70] Coles VJ, Stukel MR, Brooks MT, Burd A, Crump BC, Moran MA (2017). Ocean biogeochemistry modeled with emergent trait-based genomics. Science..

