# Supplementary material for: Metaproteomics reveal that rapid perturbations in organic matter prioritize functional restructuring over taxonomy in western Arctic Ocean microbiomes
Source: ISME J. 2019 Sep 6;14(1):39–52. doi: 10.1038/s41396-019-0503-z (PMC6908719; doi:10.1038/s41396-019-0503-z)
Supplement: Supplementary file 2 — Supplemental Methods and documentation [file 41396_2019_503_MOESM2_ESM.docx]

Supplementary Information for:

Metaproteomics reveal that rapid perturbations in organic matter inputs prioritize functional restructuring over taxonomy in Western Arctic Ocean microbiomes

Molly P. Mikan^1^, H. Rodger Harvey^1^, Emma Timmins-Schiffman^2^, Michael Riffle^3^, Damon H. May^2^, Ian Salter^4^, William S. Noble^2^, Brook L. Nunn^2*^

**Author Affiliations**

1. Ocean, Earth and Atmospheric Sciences, Old Dominion University, 406 Oceanography & Physical Sciences Building, Norfolk, VA, United States, 23529, ORCID identifiers for MPM: 0000-0002-5923-9863 & HRH: 0000-0001-7746-7758
2. Department of Genome Sciences, University of Washington, William H. Foege Hall, 3720 15th Ave NE, Seattle, WA, United States, 98195, ORCID identifiers for ET-S: 0000-0002-2478-6541, DHM: 0000-0001-6902-3153, WSN: 0000-0001-7283-4715 & BLN: 0000-0002-7361-4359
3. Department of Biochemistry, University of Washington, 1705 NE Pacific St., Seattle, ORCID identifier for MR: 0000-0003-1633-8607
4. Alfred Wegener Institute Helmholtz Center for Polar and Marine Research, Bremerhaven, Germany, ORCID identifier for IS: 0000-0002-4513-0314

*email address: [brookh@uw.edu](mailto:brookh@uw.edu) & phone: 206-616-9023

**This file includes:**

Supplementary text – Materials and Methods

References for SI citations

Legends for Figures S1 to S5

Legends for Tables S1 to S8

Legends for datasets 1-6

**Other supplementary materials for this manuscript include the following:**

Datasets 1-6

Supplementary Text – Materials and Methods

*Seawater sample collection*

Seawater was collected in August of 2013 via high volume Niskin (10 L General Oceanics equipped with CTD sensors) casts from the chlorophyll maximum in the Bering Strait (BSt) (7 m, 65° 43.44” N, 168° 57.42” W) and from the bottom waters of the Chukchi Sea (CS) (55.5 m, 72° 47.624” N, 164° 53.89” W) (Figure S1) as described in May et al. [1]. Water was collected from different depths at the two stations in order to target free-living bacterial communities that may be taxonomically distinct, due to differences in the physicochemical parameters (Figure S2). Chlorophyll a and nutrient concentration measurements (Figure S2) were collected and conducted using standard protocols (https://www.umces.edu/nutrient-analytical-services-laboratory). Upon collection, water was filtered by sequential size fractionation through 10.0 µm and 1.0 µm filters to isolate the free-living bacteria from eukaryotic grazers and large particles before incubation. Although grazers >1.0 µm were excluded from the incubations, it is possible that some grazers slipped through and that grazers <1.0 µm were present, with possible impacts on the bacterial community dynamics. For metagenomic analysis of the initial bacterial communities, a total of 7 L of 1.0 µm prefiltered water from each station was collected in 1 L aliquots onto 0.2 µm polycarbonate (PC) filters (Whatman Nuclepore), immediately frozen in liquid N_2_ and stored at -80 ºC until DNA extraction.

*Shipboard incubation set-up*

To examine bacterial community response to organic amendments, 60 L of 1.0 µm prefiltered seawater from the Bering Strait and 60 L of 1.0 µm prefiltered seawater from the Chukchi Sea were incubated shipboard for ten days at 0 ºC in the dark. This incubated temperature was a minor change for both microbiomes; *in situ* temperature at the Bering Strait station at 7 m depth was 2.06 ºC and at the Chukchi Sea, at 55.5 m depth was -1.72 ºC. The first 40 L of seawater from each station was distributed among two, 20 L carboys to act as biological replicates and supplemented with *in situ* algal organic matter (OM input) between 5.0-10.0 µm in size. This particulate organic matter (POM) was collected, filtered and then concentrated from the chlorophyll maximum of the Bering Strait. Before addition, the POM was frozen to kill cells and encourage phytoplankton cell lysis and release of bioavailable dissolved organic matter (DOM) to the bacterial community. Before subsampling, water from each biological replicate was collected, and then mixed prior to filtration. 20 L of the 1.0 µm prefiltered seawater from each station received no OM input after POM >1.0 µm was removed to examine bacterial responses to incubation conditions and residual DOM, thus functioning as the control treatment (Control). At the initial time of sampling and on days 1, 6 and 10 of the incubation experiments, a total of 1.8 L of water were passed through a 1.0 µm filter, collected onto duplicate or triplicate 0.2 µm PC filters, flash frozen in liquid nitrogen and stored at -80 °C for bacterial metaproteomics analysis. Samples for free-living bacterial phylogenetic structure (16S rRNA analysis) were collected onto 0.22 μm membrane sterivex cartridges (Millipore) on days 0, 1, 2, 4, 6 and 10 and stored at -80 °C.

*Bacterial cell counts, compound analysis and total hydrolysable amino acids*

Samples for bacterial abundance were mounted onto slides with a 0.2 µm black PC filter, fixed with 37% formalin, stained using Fluoroshield with DAPI (Sigma Aldrich) and counted using an epifluorescence microscope (Olympus Optical, Model BX50F). Whole water from each station was passed through a 10.0 µm filter before slide preparation to estimate cell counts between the sizes of 0.2-10.0 µm. A sample from each station was also passed through a 1.0 µm filter to quantify bacterial abundance 0.2-1.0 µm in size, representing bacterial concentrations at the start of the incubation experiments. The BSt preserved slide with a 1.0 µm prefilter, however, was of poor quality. Therefore, to estimate the bacterial abundance in the 0.2-1.0 µm size fraction for BSt, we applied the recovery of bacteria in the same size fraction from the CS sample. In the CS sample, 40.3% of DAPI-stained cells passed through the 1.0 µm filter compared to the 10.0 µm filter (Table S1). Therefore, assuming a similar % retention onto the 1.0 µm filter between stations, we calculated the bacterial concentration for BSt < 1.0 µm by multiplying 40.3% by the bacterial concentration < 10.0 µm from BSt. The difference in cell abundances between the 1.0 µm and 10.0 µm prefilters represents the fraction of bacteria that were retained on a 1.0 µm filter. There could be multiple reasons why we measured this difference; 1) due to bacterial cell size > 1.0 µm, 2) the bacteria were attached to material large enough to be retained, or 3) as filtration progressed, the filter clogged, trapping bacterial cells that would have otherwise passed through a 1.0 µm opening, or 4) a combination of 1-3. After initiation of the experimental setup, samples collected for bacterial abundance counts were not size fractionated and therefore those estimates represent both the free-living and POM attached bacterial fractions.

Samples for organic nitrogen (ON) and carbon (OC) and total hydrolysable amino acid (THAA) concentrations were collected onto combusted glass fiber filters (Whatman GF/F) and frozen until analysis. The microbial fraction was first passed through a 1.0 µm filter and the POM fraction was collected without prefiltration. Prior to ON and OC analysis by standard methods (https://www.umces.edu/nutrient-analytical-services-laboratory), filters were acidified in 4 M HCl solution, placed in a dessicator for 1 hour and dried at 60°C overnight. THAA analysis by gas chromatography-mass spectrometry (GC-MS) followed methods outlined in Moore et al. [2]. Briefly, Norvaline was added to each sample to act as an internal standard prior to a 4 hour acid hydrolysis with 6 M HCl at 100-110°C [3], followed by a pH-adjustment with sodium carbonate to obtain a pH range 1.5-5. Solid phase extraction and derivatization (with propyl chloroformate and propanol) was completed with an EZ:Faast method (Phenomenex). Samples were then evaporated under N_2_ gas and redissolved in an 80:20 Isooctane:Chloroform solvent. Amino acids were separated using gas chromatography (Agilent 7890A, Santa Clara, CA) with a DB-5 MS capillary column (0.25 mm ID, 30 m); oven temperature increased from 110°C to 280°C at a rate of 10°C per minute and held for 5 minutes, followed by ionization and structural identification via mass spectrometry (Agilent 5975C, Santa Clara, CA) with helium as the carrier gas. Selective Ion Monitoring was used to isolate and measure individual THAAs by identifier ions (masses provided by Phenomenex) and final quantification was made by peak integration and comparison to the internal standard.

*Metagenome and metaproteome sample preparation and sequencing*

DNA from filters collected from BSt and CS for bacterial metagenome sequencing was extracted following the protocol in Wright et al. [4]. The extraction method, plus library preparation and sequencing were identical to methods described in Timmins-Schiffman et al. [5]. Briefly, DNA was sheared to < 1 kb and desalted prior to library preparation and quality control confirmation. Sequencing of libraries from each station was completed on a one lane Illumina HiSeq 2500 (Illumina, San Diego, CA) in the Northwest Genomics Center (University of Washington). Raw sequencing reads can be accessed in NCBI’s Short Read Archive (Accession number SRP071900). The BSt and CS libraries were assembled into one combined metagenome using MOCAT, which removes contaminants, assembles reads and predicts protein sequences [6]. The final database contained 459,118 protein sequences and over 41 million unique peptide sequences.

Samples from the Chukchi Sea and Bering Strait incubation experiments were prepared and analyzed in parallel. Metaproteomic sample preparation and liquid chromatography and tandem mass spectrometry (LC-MS/MS) methods are detailed in Timmins-Schiffman et al. [5]. Briefly, cells were lysed and removed from filters using a probe sonicator (Branson Ultrasonics 250, Danbury, CT) followed by flash-freezing in liquid nitrogen. Proteins in each sample were extracted and desalted [7] prior to analysis with LC-MS/MS (nanoAcquity UPLC (Waters Corp, Milford, MA)-Q-Exactive-HF (Thermo Fisher Scientific, Waltham, MA)). Samples were analyzed in duplicate on the Q-Exactive. Mass spectrometry samples from the Chukchi Sea incubations at Day 1 with POM removed were compromised and excluded from analysis. The mass spectrometry proteomics data have been deposited to the ProteomeXchange Consortium via the PRIDE [8] partner repository with the dataset identifier PXD008780 (https://www.ebi.ac.uk/pride/archive/projects/PXD008780).

*Peptide detection and quantitation*

All database searches were performed using Comet [9] version 2015.01 rev. 2, using a concatenated decoy database in which peptide sequences were reversed but C-terminal amino acids were left in place. Search parameters included a static modification for cysteine carbamidomethylation (57.021464) and a variable modification for methionine oxidation (15.9949). Enzyme specificity was trypsin, with three missed cleavages allowed. Parent ion mass tolerance was set to 10 ppm around five isotopic peaks, and fragment ion binning was 0.02, with offset 0.0. Prior to further analysis, Comet results for technical replicates were combined. As described previously [10], to determine the full set of peptides for comparison, after each unique peptide was associated with its top-scoring spectrum irrespective of charge state, we used the Percolator algorithm [10, 11] to estimate the false discovery rate (FDR) associated with a given set of accepted peptide sequences. In this context, the FDR is defined as the proportion of the accepted peptide spectrum matches (PSMs) that are incorrect. All peptides accepted at FDR 0.01 in at least one sample were used for comparison [12]. Peptide quantitation was performed by counting the accepted PSMs for each peptide sequence.

*Taxonomic and functional annotation*

As described in Riffle et al. [13], to find the spectral counts associated with Gene Ontology (GO) molecular function, biological process and cellular component categories and associated taxonomies, a GO directed acyclic graph (DAG) and taxonomic tree were constructed for each peptide. The spectral count of each node of the graph was increased by the spectral count for the respective peptide. To do this, BLAST was first used to search all metagenome protein sequences against the UniProtKB/TrEMBL database (downloaded April 28, 2015), using the top BLAST match with an e-value less than or equal to 1E-10. Taxonomic and functional annotations of Uniprot BLAST matches were used to annotate the metagenome protein sequences.

For functional annotation of a peptide, the GO annotations of the top Uniprot BLAST match for all metagenome proteins matched by that peptide were combined and used to create a non-redundant GO DAG containing the direct GO annotations and their ancestor terms up to the root GO node. The spectral count of each of these GO terms is increased by the spectral count of the peptide. After processing all peptides, we obtain spectral counts for all GO terms that appear in the experiment and obtain a relative spectral abundance for each GO term by dividing these counts by the total number of PSMs.

Then, for each GO term, a taxonomic analysis was performed to find the contribution to that GO term by each taxon. For each peptide matched to a GO term, the taxonomic annotations of the best Uniprot BLAST match (e-value less than or equal to 1E-10) for all metagenome proteins matched by that peptide were used to determine the lowest common ancestor (LCA) associated with the peptide (calculated using Unipept [14, 15] and an in-house Python script). The GO term’s spectral count for the LCA and all ancestor terms was increased by the spectral count of the respective peptide. After processing all peptides for this GO term, we divided each taxon spectral count by the total spectral count for this GO term to obtain the unambiguous, relative contribution of each taxon to the spectral count for this GO term. At most, the relative contribution of all taxa at the same taxonomic level (e.g., class) will add up to 1, if all peptides for that GO term resulted in a LCA at the class level or more granular. If some peptides were matched to an LCA less granular than class (e.g., phylum) the relative contributions at the class level will add up to less than 1. We report unambiguous taxonomic classification per GO function at the class level. If the taxa at the class level for a GO term summed to a relative contribution less than one (some peptides had a LCA at a less granular classification (e.g., phylum or kingdom) or had no taxonomic information), the difference was assigned to the Unclassified taxonomic category and a PSM count was assigned. Eight non-bacterial classes were detected and were removed from any further analysis, as they were not the focus of this research. All source code for calculating GO spectral counts, taxonomic analysis, and comparing results between samples is available at https://github.com/metagomics/mmikan-metaproteomics-2018.

*Gene Ontology (GO) enrichment analysis*

An enrichment analysis of GO functions was performed using in-house programs, as described by Riffle et al. (MetaGOmics: <https://www.yeastrc.org/metagomics/>) [13]. Briefly, each pair of mass spectrometry runs was compared against one another by first performing Laplace-correction for all GO term spectral counts in each run by adding 1 to the spectral count for each GO term that appeared in any run (so that a log-fold analysis may be performed for GO terms that do not appear in a given run). The relative spectral abundance was calculated by dividing this count by the total spectra in that run (plus the number of GO terms). Log_2_ fold changes of PSM ratios were calculated between two samples for each GO function. For this study, we compared sequential time points within each experiment (i.e., BSt compared to day 1, day 1 compared to day 6, and day 6 compared to day 10). Terminal GO terms with a two-tailed test of proportions p-value < 0.01 (Bonferroni corrected) was considered significant, and was included in the enrichment analysis results. Source code used to conduct these analyses is available at https://github.com/metagomics/mmikan-metaproteomics-2018.

*16S rRNA: DNA Extraction and Amplicon Sequencing*

Methods for 16S rRNA isolation and amplicon sequencing followed Fadeev et al. [16]. Briefly, genomic bacterial DNA was isolated from both filter membranes in a combined chemical and mechanical procedure using the PowerWater DNA Isolation Kit (MO BIO Laboratories, Inc., Carlsbad, CA, USA). Prior to DNA isolation the sterivex cartridges of the 0.22 μm membranes were cracked open in order to place the filters in the kit-supplied bead beating tubes. The isolation was continued according to the manufacturer’s instructions, and DNA was stored at − 20°C.  Library preparation was performed according to the standard instructions of the 16S Metagenomic Sequencing Library Preparation protocol (Illumina, Inc., San Diego, CA, USA). The hypervariable V3–V4 region of the bacterial 16S was amplified using bacterial primers S-D-Bact-0341-b-S-17 (5`-CCTACGGGNGGCWGCAG-3`) and S-D-Bact-0785-a-A-21 (5`-GACTACHVGGGTATCTAATCC-3`) [17]. Sequences were obtained on the Illumina MiSeq platform in a 2 × 300 bp paired-end run as well as in a 2 × 250 bp paired-end run on the Illumina HiSeq platform (CeBiTec Bielefeld, Germany).

## *16S rRNA: Bioinformatic and Statistical Analyses*

Methods for 16S rRNA sequencing followed methods detailed in Fadeev et al. [16]. Briefly, the raw paired-end reads were primer-trimmed using cutadapt [18], quality trimmed using trimmomatic v0.32 (with sliding window of four bases and a minimum average quality of 15) [19] and merged using PEAR v0.9.5 [20]. Clustering into OTUs was done with Swarm algorithm using default parameters (v2.0) [21]. One representative sequence per OTU was taxonomically classified using SINA (SILVA Incremental Aligner; v1.2.11; Silva reference database release 128) at a minimum alignment similarity of 0.9, and a last common ancestor consensus of 0.7 [22]. OTU which were not taxonomically assigned to Bacteria or occurred with only a single sequence in the whole data set were excluded from further analysis. The entire statistical analysis was conducted using R (v3.4.1 ; http://www.Rproject.org/) in RStudio (v1.0.153) [23]. Pearson correlation (*rcorr* function, Hmisc package in R) was used to test for linear correlation of relative abundance data between genera composing >5% of total abundances.

*Hierarchical clustering*

Dissimilarity between variables (GO terms that changed significantly over time) was performed with the *dist* function in R, and was measured with the Euclidean distance metric. Hierarchical clustering was performed using the *hclust* function in R with the complete linkage method, which maximizes the dissimilarity between merged variables and the rest of the variables, represented as node height. Cutting the dendrograms at a height of 3 (h=3) resulted in 7 clusters for the BSt functions (Table S7) and 5 clusters for the CS functions (Table S8) that changed over time. Heatmaps of the log_2_ fold changes between consecutive time points for the GO terms were created using the *heatmap.2* function in gplot package in R. Functional changes below a log_2_ fold change threshold of 0.15 were excluded from analysis and included three terms with significant change between day 6 and day 10 of the BSt OM input experiment (functions (with log_2_ fold changes): organic substance (-0.1175), primary (-0.1193) and cellular metabolic processes (-0.1179)).

*References for SI citations*

1. May DH, Timmins-Schiffman E, Mikan MP, Haryey HR, Borenstein E, Nunn BL, et al. An Alignment-Free "Metapeptide" Strategy for Metaproteomic Characterization of Microbiome Samples Using Shotgun Metagenomic Sequencing. J Proteome Res. 2016;15(8):2697-705.

2. Moore EK, Nunn BL, Goodlett DR, Harvey HR. Identifying and tracking proteins through the marine water column: insights into the inputs and preservation mechanisms of protein in sediments. Geochim Cosmochim Acta. 2012;83:324-59.

3. Cowie GL, Hedges JI. Improved amino-acid quantification in environmental-samples - Charge-matched recovery standars and reduced analysis time. Mar Chem. 1992;37(3-4):223-38.

4. Wright JJ, Lee S, Zaikova E, Walsh DA, Hallam SJ. DNA extraction from 0.22 microM Sterivex filters and cesium chloride density gradient centrifugation. J Vis Exp. 2009(31).

5. Timmins-Schiffman E, May DH, Mikan M, Riffle M, Frazar C, Harvey HR, et al. Critical decisions in metaproteomics: achieving high confidence protein annotations in a sea of unknowns. ISME J. 2017;11(2):309-14.

6. Kultima JR, Sunagawa S, Li J, Chen W, Chen H, Mende DR, et al. MOCAT: a metagenomics assembly and gene prediction toolkit. PLoS One. 2012;7(10):e47656.

7. Nunn BL, Slattery KV, Cameron KA, Timmins‐Schiffman E, Junge K. Proteomics of Colwellia psychrerythraea at subzero temperatures–a life with limited movement, flexible membranes and vital DNA repair. Environ Microbiol. 2015;17(7):2319-35.

8. Vizcaino JA, Csordas A, del-Toro N, Dianes JA, Griss J, Lavidas I, et al. 2016 update of the PRIDE database and its related tools (vol 44, pg D447, 2016). Nucleic Acids Res. 2016;44(22):11033-.

9. Eng JK, Jahan TA, Hoopmann MR. Comet: An open‐source MS/MS sequence database search tool. Proteomics. 2013;13(1):22-4.

10. Granholm V, Navarro JF, Noble WS, Käll L. Determining the calibration of confidence estimation procedures for unique peptides in shotgun proteomics. J Proteomics. 2013;80:123-31.

11. Kall L, Canterbury JD, Weston J, Noble WS, MacCoss MJ. Semi-supervised learning for peptide identification from shotgun proteomics datasets. Nat Methods. 2007;4(11):923-5.

12. Elias JE, Gygi SP. Target-decoy search strategy for increased confidence in large-scale protein identifications by mass spectrometry. Nat Methods. 2007;4(3):207-14.

13. Riffle M, May DH, Timmins-Schiffman E, Mikan MP, Jaschob D, Noble WS, et al. MetaGOmics: A Web-Based Tool for Peptide-Centric Functional and Taxonomic Analysis of Metaproteomics Data. Proteomes. 2018;6(1).

14. Mesuere B, Debyser G, Aerts M, Devreese B, Vandamme P, Dawyndt P. The Unipept metaproteomics analysis pipeline. Proteomics. 2015;15(8):1437-42.

15. Mesuere B, Devreese B, Debyser G, Aerts M, Vandamme P, Dawyndt P. Unipept: Tryptic Peptide-Based Biodiversity Analysis of Metaproteome Samples. J Proteome Res. 2012;11(12):5773-80.

16. Fadeev E, Salter I, Schourup-Kristensen V, Nöthig E-M, Metfies K, Engel A, et al. Microbial Communities in the East and West Fram Strait During Sea Ice Melting Season. Front Mar Sci. 2018;5(429).

17. Klindworth A, Pruesse E, Schweer T, Peplies J, Quast C, Horn M, et al. Evaluation of general 16S ribosomal RNA gene PCR primers for classical and next-generation sequencing-based diversity studies. Nucleic Acids Res. 2013;41(1):e1-e.

18. Martin M. Cutadapt removes adapter sequences from high-throughput sequencing reads. EMBnet J. 2011;17(1):pp. 10-2.

19. Bolger AM, Lohse M, Usadel B. Trimmomatic: a flexible trimmer for Illumina sequence data. Bioinformatics. 2014;30(15):2114-20.

20. Zhang J, Kobert K, Flouri T, Stamatakis A. PEAR: a fast and accurate Illumina Paired-End reAd mergeR. Bioinformatics. 2014;30(5):614-20.

21. Mahé F, Rognes T, Quince C, de Vargas C, Dunthorn M. Swarm: robust and fast clustering method for amplicon-based studies. PeerJ. 2014;2:e593.

22. Pruesse E, Peplies J, Glöckner FO. SINA: accurate high-throughput multiple sequence alignment of ribosomal RNA genes. Bioinformatics. 2012;28(14):1823-9.

23. Studio R. RStudio: integrated development environment for R. RStudio Inc, Boston, Massachusetts. 2012.

*Legends for Figures S1 to S5*

Figure S1. Map showing locations of water sampling from the Bering Strait (BSt; 7m; 65° 43.44” N, 168° 57.42” W) and the Chukchi Sea (CS; 55.5 m; 72° 47.624” N, 164° 53.89” W).

Figure S2. Salinity, Temperature, Chlorophyll a and nutrient concentrations from the water column of the Bering Strait (BSt) (solid line) and Chukchi Sea (CS) stations (dashed line). Water was collected from 7 m in the BSt (integrated chlorophyll a: 226.88 mg/m^2^) and 55.5 m from the CS (integrated chlorophyll a: 2.64 mg/m^2^). Data was provided by Lee Cooper: <http://arcticstudies.org/hannashoal/data.html>.

Figure S3. Distribution of taxonomic assignments that can be reported for all peptide spectrum matches (PSMs) passing confidence threshold for A) Bering Strait and B) Chukchi Sea metaproteomic data. Note that in both figures, more PSMs are assigned a species-level designation than a genus level designation. This is counterintuitive and results from the inconsistencies found within the taxonomic databases. Many taxonomic assignments are missing genus-level information and because this is an automated data processing, manual interpretations were not completed to retain reproducibility.

Figure S4. Temporal bacterial contributions to community structure in the Bering Strait (BSt) and Chukchi Sea (CS) microbiomes under Control and algal OM input. Genera with at least 5% of relative abundance at any time within the experiments were represented, and otherwise were combined into the ‘Other’ category comprising 347 genera. Genera *Polaribacter* spp. and *Owenweeksia* spp. belong to Class Flavobacteria; Genera *Balneatrix* spp., unclassified *Oceanospirillales* spp., unclassified *Colwelliaceae* spp., *Colwellia* spp., *Pseudoalteromonas* spp., SAR92 clade and *Acinetobacter* spp. belong to Class Gammaproteobacteria; Genera unclassified *Pelagibacter* spp. (SAR11 clade) and *Sulfitobacter* spp. belong to Class Alphaproteobacteria. All data are included in Dataset 6.

Figure S5. Heatmap of Chukchi Sea (CS) Gene Ontology (GO) functions with significant peptide spectrum matches (PSM) log_2_ fold changes (Bonferroni-corrected p-value < 0.01 from a two-tailed test of proportions) between time points with algal organic matter input (aPOM): column 1: initial (day 0) CS microbiome sample compared to day 1, column 2: day 1 to day 6, column 3: day 6 to day 10. Color shading indicates the degree of log_2_ fold change as seen in the Color Key. Functions with log_2_ fold changes are outlined in Table S8.

*Legends for Tables S1 to S8*

Table S1. Bering Strait (BSt) and Chukchi Sea (CS) bacterial and particulate organic matter (POM) measurements from the 10 day incubation experiments. BDL = below detection limit. Dark grey cell = estimated value (see details in SI Materials & Methods). OM input = algal organic matter input; Control = control treatment where POM >1.0 µm was removed without OM input. THAA = total hydrolysable amino acids. ON = organic nitrogen; OC = organic carbon. PC = 0.2 µm polycarbonate filters (Whatman Nuclepore). GF/F = glass fiber filters (Whatman).

Table S2. List of non-bacterial taxonomic classes that were assigned to peptides in the A) Bering Strait (BSt) and B) Chukchi Sea (CS) metaproteomes. BSt and CS = initial bacterial community sample. OM = algal organic matter input treatment; Control = particulate organic matter removal control.

**Table S3.** Calculated rates of change for functionality assignments in peptide data and taxonomic assignments in peptide data for OM Input and Control incubations. Calculations, raw PSM ratio data and python script used to permute data 10,000 times for null hypothesis distribution included in Dataset 5.

Table S4. Bacterial taxonomic phyla and classes for A) Bering Strait (BSt) and B*)* Chukchi Sea (CS) with peptide assignments in the metaproteomic dataset. x = presence; n.d. = no mass spectrometry data collected; blank = class not present at that time; Initial bacterial community samples = BSt and CS; OM input = algal organic matter input; Control = substrate limitation within the particulate organic matter (POM) removal control.

**Table S5.** Bacterial taxonomic phyla and classes for A) Bering Strait (BSt) and B) Chukchi Sea (CS) with operational taxonomic units (OTUs) in the 16S rRNA dataset. x = presence; blank = class not present at that time; Initial bacterial community samples = BSt and CS; OM input = algal organic matter input; Control = substrate limitation within the particulate organic matter removal control. Uncl = unclassified.

Table S6. Total number of peptides, peptide spectrum matches (PSM), PSMs that match Gene Ontology (GO) terms, and total GO terms per time point for A) Bering Strait (BSt) and B) Chukchi Sea (CS) bacterial incubations. BSt and CS = initial bacterial community sample. OM input = algal organic matter input; Control = control treatment where particulate organic matter (POM) >1.0 µm was removed without OM input. n.d. = no data (mass spectrometry samples from the Chukchi Sea incubations at day 1 within the control were compromised and excluded from analysis).

**Table S7.** Gene Ontology (GO) function A) log_2_ fold changes and B) peptide spectrum matches (PSM) that changed significantly (Bonferroni-corrected p-value < 0.01 from a two-tailed test of proportions) over time from the Bering Strait (BSt) incubations. A negative value = a decrease over time. BSt represents the initial microbiome. OM = algal organic matter inputs; Control = particulate organic matter removal as control treatment.

Table S8. Gene ontology (GO) functions from the Chukchi Sea (CS) incubations that changed significantly (Bonferroni-corrected p-value < 0.01 from a two-tailed test of proportions) over time and their log_2_ fold changes under algal organic matter input (OM input). A negative value = a decrease over time. CS represents the initial microbiome.

*Legends for datasets 1-6*

**Datasets 1-4.** Each Excel Workbook file represents the metaproteomic data collected for all time points (initial, T0, T1, T6 & T10; separated by worksheets within workbook) from a particular experimental incubation (i.e., Bering St OM, ChukSea OM, Bering St particulate organic matter control (Control), ChukSea Control). Each worksheet contains the total number of spectra that correlate to each Gene Ontology term as broken down by taxonomic level. Column headers: GO Accession: Gene Ontology accession number (e.g., GO:0016887), GO Name: given name of the Gene Ontology category (e.g., ATPase activity), GO Aspect: 1 of 3 GO broad categories: molecular function, biological process, cellular component, Taxonomy ID: Uniprot defined taxonomic identification number (i.e., 135619 = Oceanospirillaceae), Taxonomy Name: Uniprot defined taxonomic name at defined taxonomic rank (i.e., Oceanospirillaceae; rank= order), Taxonomy Rank: taxonomic rank, Taxonomy PSM Count: total number of peptide spectral matches that correlate to defined gene ontology term at the defined taxonomic level (rank) (i.e., integers 1-*n*), Taxonomy PSM Ratio: the ratio of PSMs for the defined GO term at the specified taxonomic rank to the total number of PSMs for all taxonomic ranks (i.e., <1). The taxonomic name “root” is a term that indicates it represents all taxonomic levels (superkingdom through species) and is listed as “no rank” under Taxonomic rank. Example: ATPase activity has 91 PSMs at the no rank Taxonomic rank, and of those, 12 PSMs correlate to Oceanospirillaceae (rank= order). The Taxonomy PSM ratio for Oceanospirillaceae is 12/91 = 0.14, or 14% of the ATPase activity peptide spectral matches can be correlated to the order Oceanospirillaceae. Unambiguous taxonomic classification per GO function at each taxonomic level is reported. Some peptides had a least common ancestor assignment at a less granular classification or had no taxonomic information; when the sum of PSM Ratios for any taxonomic level (e.g., class) per function was less than 1, the difference makes up the Unclassified taxonomic category.

**Dataset 5.** There are four Excel Worksheets within the workbook file that correspond to the extract ratio of peptide spectral matches from each timepoint from the different incubations. There are 4 workbooks with the calculations for : ChS_Goterm_functional_rate_change, ChS_Goterm_taxonomic_rate_change, BST_Goterm_functional_rate_change, BSt_Goterm_taxonomic_rate_change. These worksheets include:

initial, T1, T6 =Ratio of Peptide spectral Matches that are assigned to the row name (either Taxonomic class or functional GO term. Terms d1 and d6 are the differences between initial timepoint PSM ratio and T1 PSM ratio, and the difference between T6 and T1. dsum = the sum of these differences. r1 and r2 are the calculated rate of change in those ratios: r1 = d1/ 1 day; r2 = d2/5 days. rsum = the sum of the rates for a given taxa or function. Mean sum of the rate of change is determined from all rsum values from each incubation condition and or Go category (in the case for taxonomic changes. The final worksheet “PythonScript_permutations” is the python script used to determine if the taxonomic rate of change was significantly different from the functional rate of change within each incubation. The matrices of functional PSM ratios were permuted (at the row level) 10,000 times to empirically estimate a null distribution and used to calculate a p-value for observing the observed difference in rates.

**Dataset 6.** 16S rRNA sequence results to the level of genus for all samples. BSt = Bering Strait microbiome; CS = Chukchi Sea microbiome; Days 0-10 (T0, T1, T2, T4, T6, T10); Organic matter perturbations (Control= particulate organic matter removal; OM = algal organic matter input).
