# Supplementary figures and images for: Metaproteomics reveal that rapid perturbations in organic matter prioritize functional restructuring over taxonomy in western Arctic Ocean microbiomes
Source: ISME J. 2019 Sep 6;14(1):39–52. doi: 10.1038/s41396-019-0503-z (PMC6908719; doi:10.1038/s41396-019-0503-z)

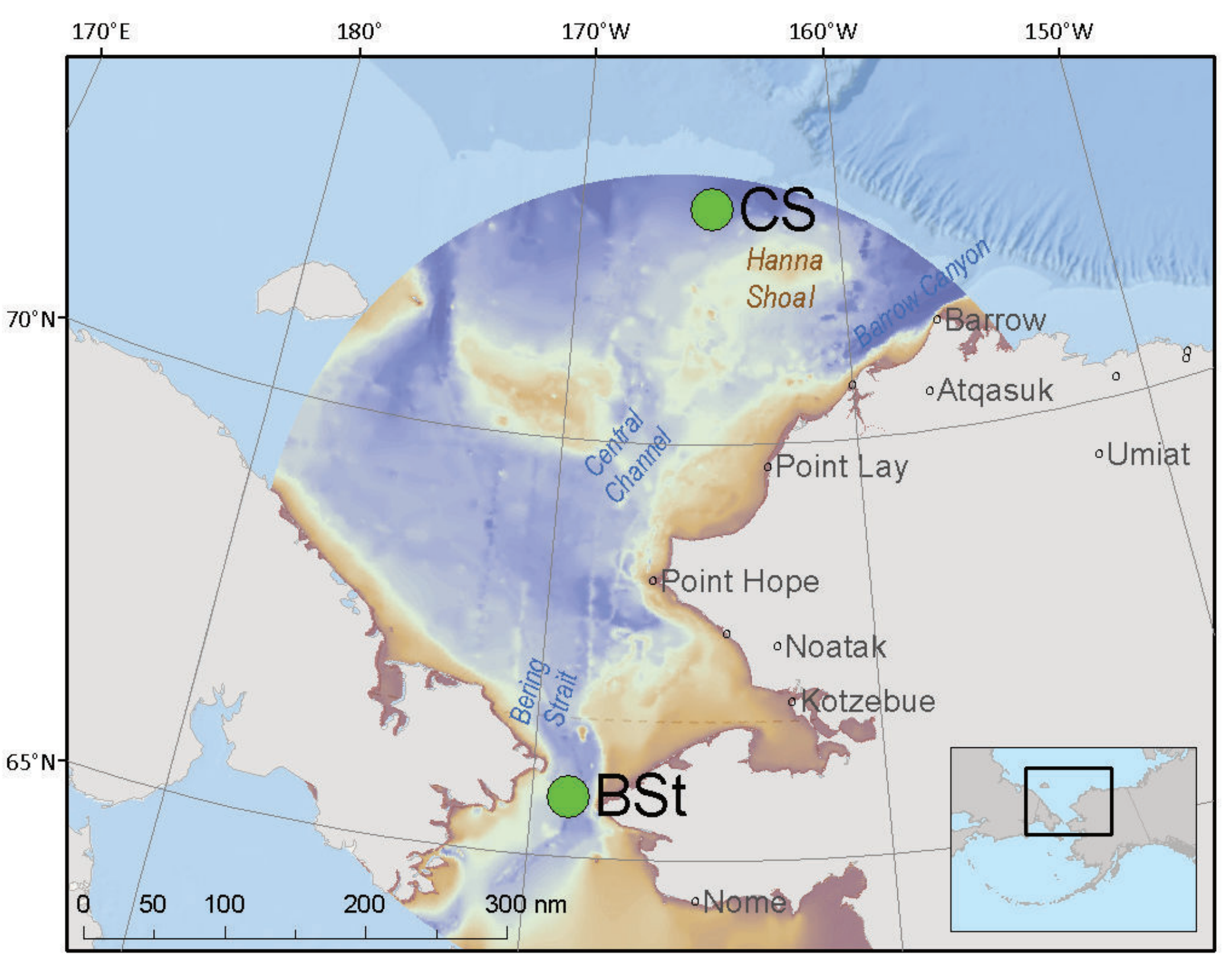

Supplement: Supplementary file 3 — Figure S1 [file 41396_2019_503_MOESM3_ESM.tif]

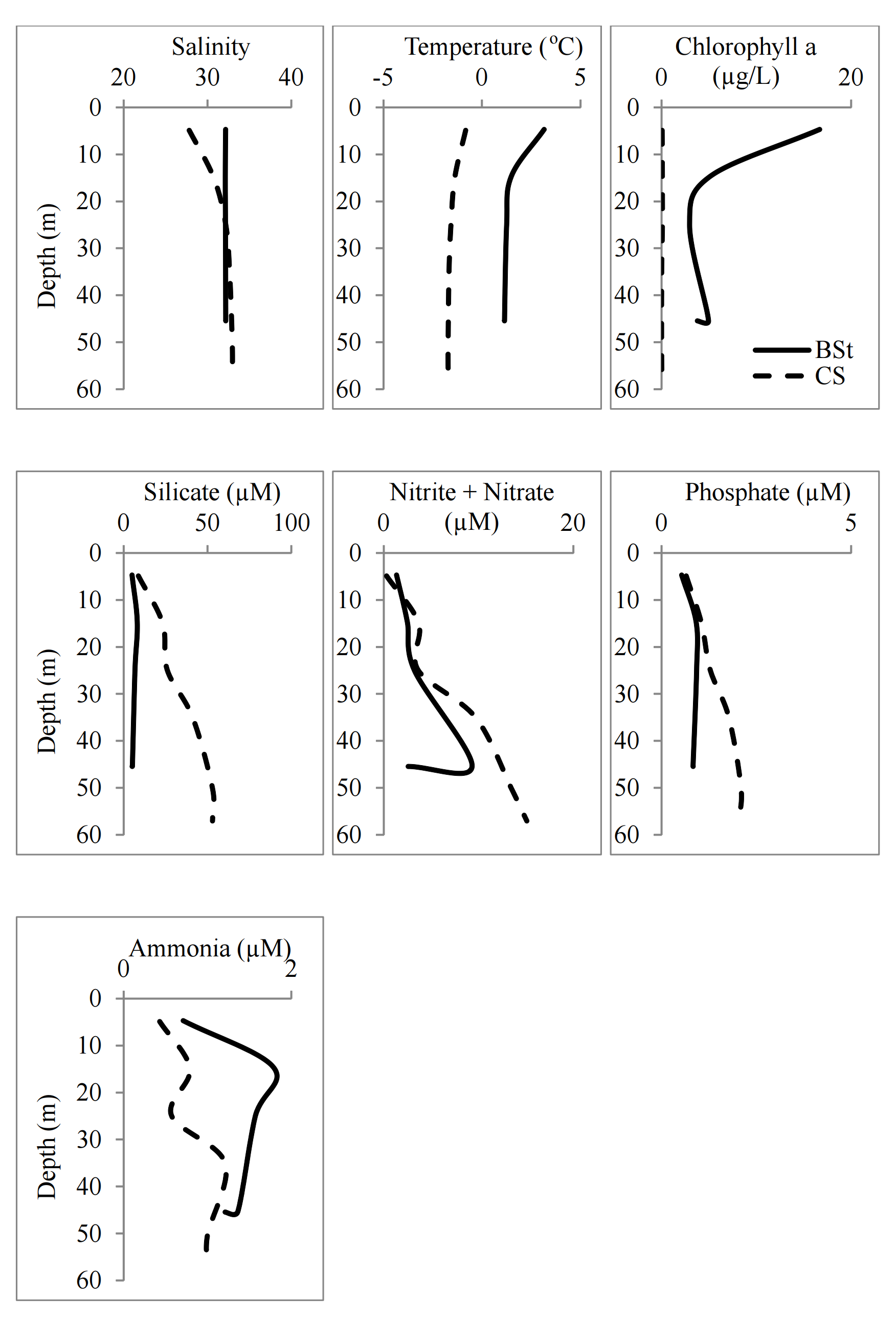

Supplement: Supplementary file 4 — Figure S2 [file 41396_2019_503_MOESM4_ESM.tif]

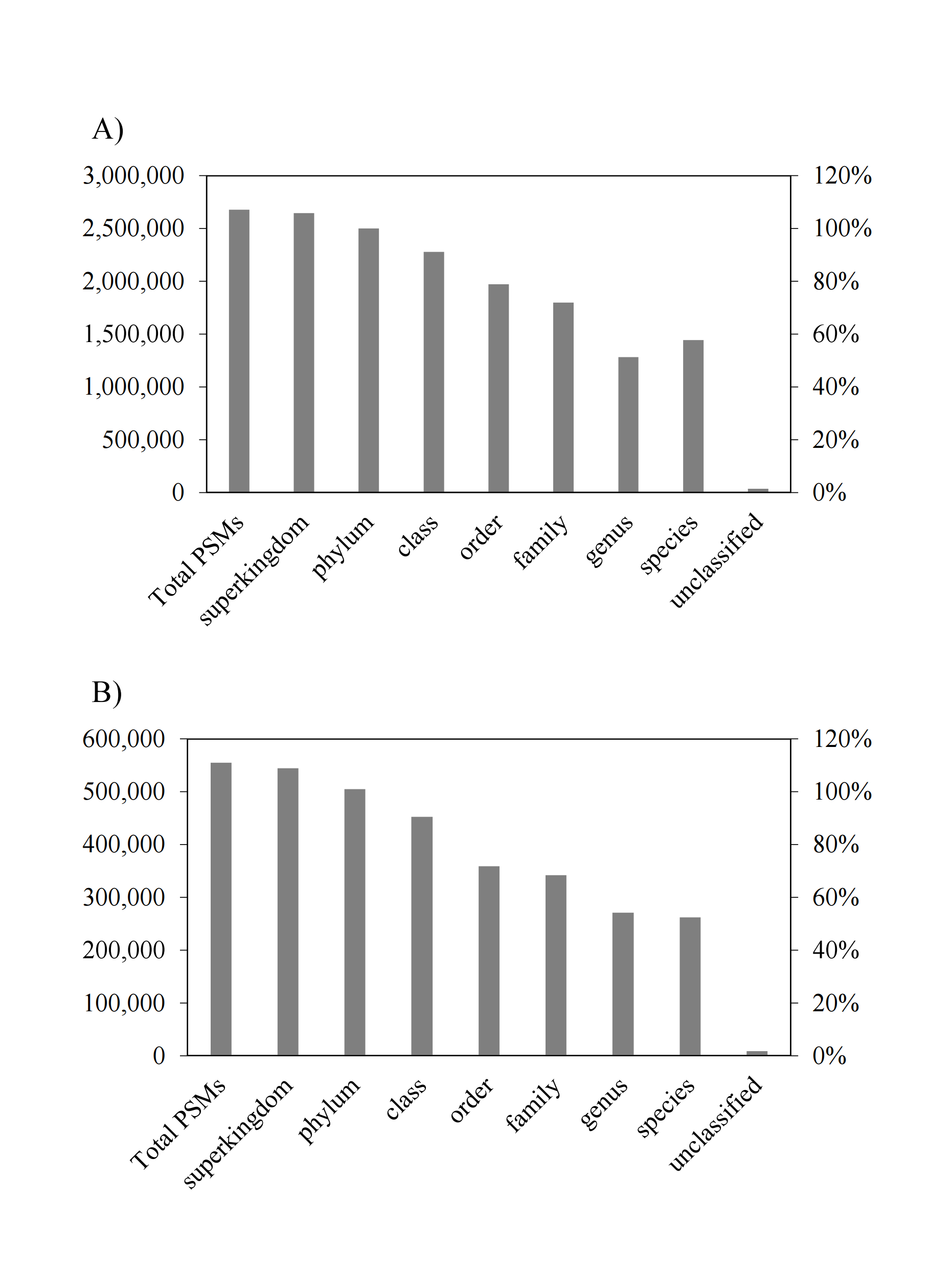

Supplement: Supplementary file 5 — Figure S3 [file 41396_2019_503_MOESM5_ESM.tif]

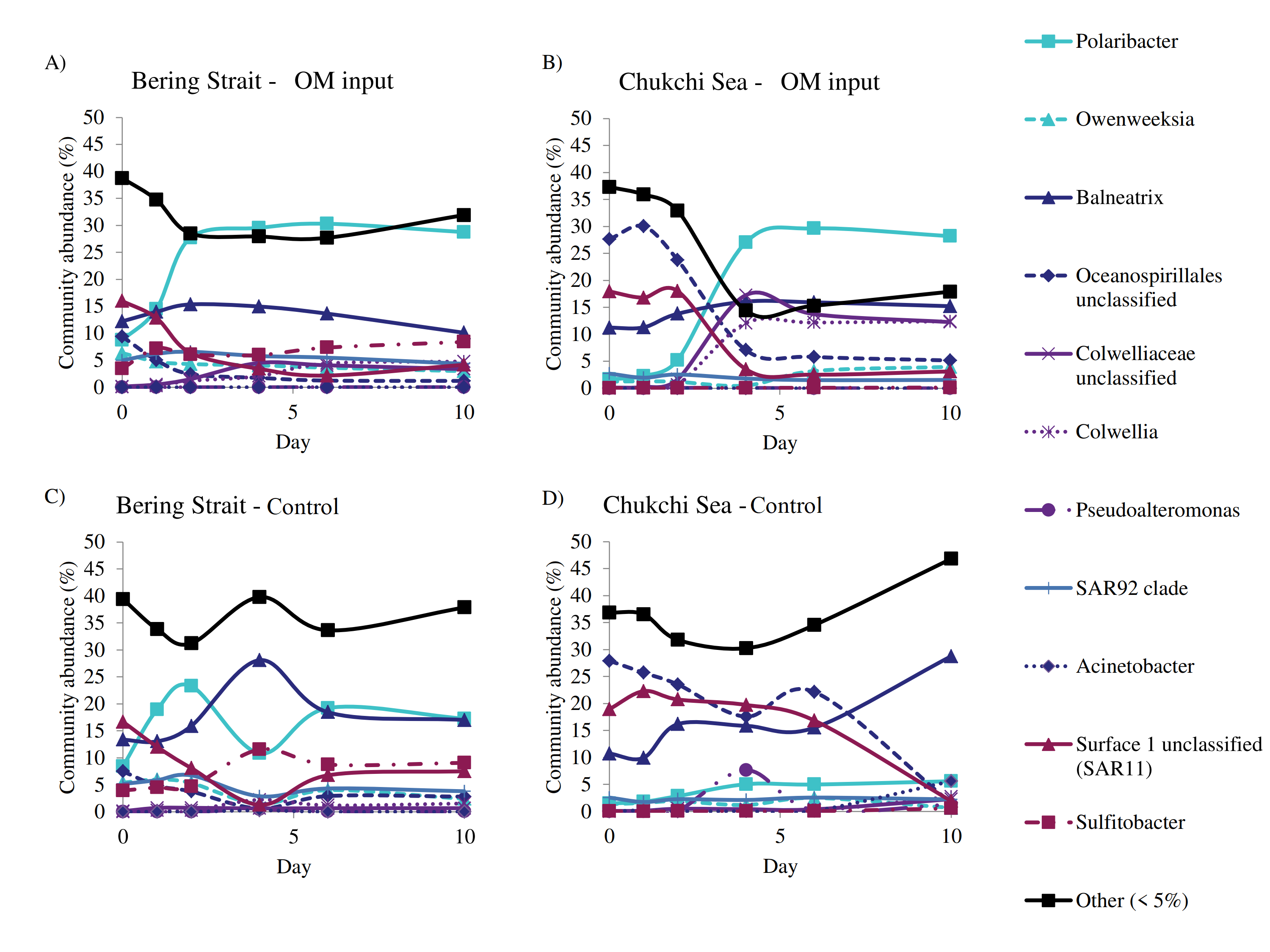

Supplement: Supplementary file 6 — Figure S4 [file 41396_2019_503_MOESM6_ESM.tif]

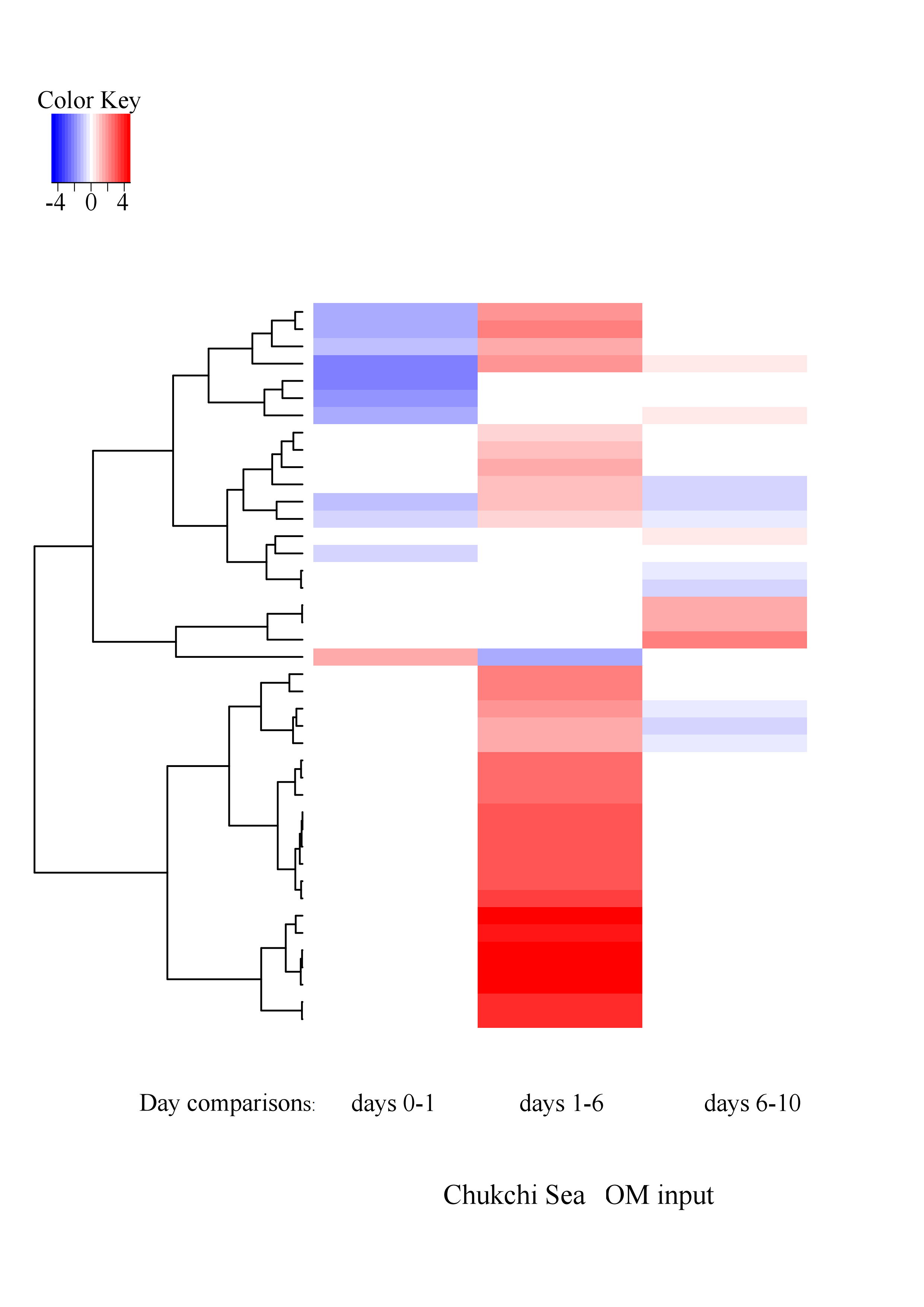

Supplement: Supplementary file 7 — Figure S5 [file 41396_2019_503_MOESM7_ESM.tif]
