# Supplementary material for: Metaproteomics reveal that rapid perturbations in organic matter prioritize functional restructuring over taxonomy in western Arctic Ocean microbiomes
Source: ISME J. 2019 Sep 6;14(1):39–52. doi: 10.1038/s41396-019-0503-z (PMC6908719; doi:10.1038/s41396-019-0503-z)
Supplement: Supplementary file 10 — Table S3 [file 41396_2019_503_MOESM10_ESM.docx]

Table S3. Calculated rates of change for functionality assignments in peptide data and taxonomic assignments in peptide data for OM Input and Control incubations.

|  |  | **Bering Strait Incubations** | | | | **Chukchi Sea Incubations** | | | |
| --- | --- | --- | --- | --- | --- | --- | --- | --- | --- |
|  |  | **Rate of Change in Functionality** | **Rate of Change for taxonomy** | | | **Rate of Change in Functionality** | **Rate of Change for taxonomy** | | |
|  |  | *across all classes* | *across molecular function GO terms* | *across cellular component GO terms* | *across biological process GO terms* | *across all classes* | *across molecular function GO terms* | *across cellular component GO terms* | *across biological process GO terms* |
| **OM INPUT** | Mean rate change in PSM ratios | 0.0235 | 0.0076 | 0.0082 | 0.0082 | 0.0208 | 0.0195 | 0.0180 | 0.0209 |
|  | Statistical test if Taxonomic rate of change is significantly less than Functional rate of change* |  | n=71, p<.0001 | n=71, p<.0001 | n=71, p<.0001 |  | n= 71, 0.018 | n= 71, 0.0196 | n = 71, 0.403 |
| **CONTROL** | Mean rate change in PSM ratios | 0.0143 | 0.0072 | 0.0080 | 0.0078 | N/A | N/A | N/A | N/A |
|  | Statistical test if Taxonomic rate of change is significantly less than Functional rate of change* |  | n=71, p<.0001 | n=71, p<.0001 | n=71, p<.0001 |  | N/A | N/A | N/A |

* n : number of instances tested over 10,000 permutations, calculated p-value
