# Supplementary material for: Metaproteomics reveal that rapid perturbations in organic matter prioritize functional restructuring over taxonomy in western Arctic Ocean microbiomes
Source: ISME J. 2019 Sep 6;14(1):39–52. doi: 10.1038/s41396-019-0503-z (PMC6908719; doi:10.1038/s41396-019-0503-z)
Supplement: Supplementary file 14 — Table S7a [file 41396_2019_503_MOESM14_ESM.pdf]

A)

Cluster #	Function code	GO function	GO category	BSt to day 1 OM input	day 1 to day 6 OM input	BSt to day 1 Control	day 1 to day 6 Control
2 - Protein synthesis & ATP synthase activity	1	large ribosomal subunit rRNA binding	mf	1.59			
	2	regulation of translation	bp	2.85			
	3	peptidyl-prolyl cis-trans isomerase activity	mf	1.46		1.27	
	4	protein peptidyl-prolyl isomerization	bp	1.47		1.28	
	5	small ribosomal subunit	cc	1.13	-0.94	0.86	-0.67
	6	translation	bp	0.99	-0.88	0.69	-0.65
	7	structural constituent of ribosome	mf	1.72	-1.74	0.79	-0.77
	8	ribosome	cc	0.96	-1.82	0.68	-0.79
	9	large ribosomal subunit	cc	0.87	-1.25	0.72	-0.57
	10	tRNA binding	mf	1.47	-0.97	0.96	-0.66
	11	unfolded protein binding	mf		-0.68		-0.57
	12	protein folding	bp	0.37	-0.61		-0.45
	13	proton-transporting ATP synthase activity, rotational mechanism	mf		-0.56		
	14	plasma membrane ATP synthesis coupled proton transport	bp		-0.50		
	15	proton-transporting ATP synthase complex, coupling factor F(o)	cc		-1.37		
	16	DNA-templated transcription, termination	bp		-1.99		
	17	rRNA binding	mf		-1.86	0.99	
	18	intracellular	cc	0.74	-0.89	0.45	
1	19	glycolytic process	bp	3.53			
6 - amino acid synthesis & energy conversion	20	transferase activity, transferring acyl groups	mf				1.44
	21	glutamine family amino acid biosynthetic process	bp				1.70
	22	coenzyme binding	mf				0.93
	23	ligase activity, forming carbon-nitrogen bonds	mf				0.92
	24	ligase activity, forming carbon-sulfur bonds	mf				1.34
	25	dicarboxylic acid metabolic process	bp				1.28
	26	valine biosynthetic process	bp				1.68
	27	isoleucine biosynthetic process	bp				1.59
	28	oxidoreductase activity, acting on the CH-NH2 group of donors	mf				1.79
	29	serine family amino acid metabolic process	bp				2.48
	30	ketol-acid reductoisomerase activity	mf		1.66		1.72
	31	ATP-binding cassette (ABC) transporter complex	cc		1.88		1.55
	32	4 iron, 4 sulfur cluster binding	mf		1.30		1.68
3 - translation binding & carbohydrate energy conversion	33	membrane	cc				-0.16
	34	transport	bp	-0.36			
	35	RNA binding	mf				-0.58
	36	nucleoside-triphosphatase activity	mf	0.39			
	37	protein binding	mf	0.45			
	38	translation factor activity, RNA binding	mf	0.55			
	39	ATP binding	mf	0.27			
	40	alpha-amino acid metabolic process	bp			0.98	
	41	receptor activity	mf	-0.76	0.44		-0.23
	42	cell outer membrane	cc	-0.88			
	43	lyase activity	mf		0.84		
	44	monocarboxylic acid metabolic process	bp		0.72		
	45	metal ion binding	mf		0.57		
	46	oxidoreductase activity, acting on the aldehyde or oxo group of donors	mf		1.19		
	47	tricarboxylic acid cycle	bp		1.44		1.74
	48	oxidation-reduction process	bp		0.72		0.98
4 - nitrogen reallocation & vitamin B synthesis	49	glutamate-ammonia ligase activity	mf		1.83		
	50	pyridoxal phosphate binding	mf		1.82		
	51	glutamine biosynthetic process	bp		1.85		
	52	enzyme regulator activity	mf		1.99		
	53	nitrogen compound transport	bp		1.99		
	54	nitrogen fixation	bp		1.59		
	55	formate-tetrahydrofolate ligase activity	mf		2.53		
	56	folic acid-containing compound biosynthetic process	bp		2.57		
	57	tetrahydrofolate metabolic process	bp		2.26		
	58	glutamate synthase (NADPH) activity	mf		3.38		
	59	regulation of nitrogen utilization	bp		3.42		
	60	glutamate biosynthetic process	bp		3.17		
	61	thiamine biosynthetic process	bp		3.75		
5 - energy conversion & carbohydrate metabolism	62	oxidoreductase activity, acting on NAD(P)H, quinone or similar compound as acceptor	mf			2.99	
	63	nicotinamide nucleotide metabolic process	bp			2.90	
	64	nucleoside diphosphate phosphorylation	bp			2.54	
	65	metal ion transport	bp			3.77	
	66	single-organism catabolic process	bp		1.22	2.36	
	67	single-organism carbohydrate metabolic process	bp		0.96	2.18	
7 - formate & viral activity	68	molybdenum ion binding	mf	-2.13		-1.73	2.17
	69	formate dehydrogenase (NAD+) activity	mf	-2.99		-2.21	2.26
	70	outer membrane-bounded periplasmic space	cc	-1.45	0.89	-0.94	1.18
	71	viral capsid	cc	-2.64		-2.55	
