# Supplementary material for: Metaproteomics reveal that rapid perturbations in organic matter prioritize functional restructuring over taxonomy in western Arctic Ocean microbiomes
Source: ISME J. 2019 Sep 6;14(1):39–52. doi: 10.1038/s41396-019-0503-z (PMC6908719; doi:10.1038/s41396-019-0503-z)
Supplement: Supplementary file 15 — Table S7b [file 41396_2019_503_MOESM15_ESM.pdf]

B)

Cluster #	Function code	GO function	GO category	BSt	day 1 OM input	day 6 OM input	day 1 Control	day 6 Control
2 - Protein synthesis & ATP synthase activity	1	large ribosomal subunit rRNA binding	mf	3.31E-03	9.97E-03	0.00E+00	0.00E+00	0.00E+00
	2	regulation of translation	bp	1.77E-03	7.50E-03	0.00E+00	0.00E+00	0.00E+00
	3	peptidyl-prolyl cis-trans isomerase activity	mf	5.62E-03	1.54E-02	0.00E+00	1.35E-02	0.00E+00
	4	protein peptidyl-prolyl isomerization	bp	5.46E-03	1.52E-02	0.00E+00	1.33E-02	0.00E+00
	5	small ribosomal subunit	cc	1.85E-02	3.97E-02	2.08E-02	3.23E-02	2.03E-02
	6	translation	bp	1.45E-01	2.89E-01	1.57E-01	2.35E-01	1.50E-01
	7	structural constituent of ribosome	mf	1.19E-01	2.50E-01	1.19E-01	2.06E-01	1.21E-01
	8	ribosome	cc	1.43E-01	2.78E-01	1.32E-01	2.29E-01	1.33E-01
	9	large ribosomal subunit	cc	2.51E-02	4.60E-02	2.26E-02	4.08E-02	2.75E-02
	10	tRNA binding	mf	1.40E-02	3.73E-02	1.90E-02	2.74E-02	1.74E-02
	11	unfolded protein binding	mf	0.00E+00	7.76E-02	5.09E-02	5.99E-02	4.04E-02
	12	protein folding	bp	7.31E-02	9.43E-02	6.17E-02	7.39E-02	5.40E-02
	13	proton-transporting ATP synthase activity, rotational mechanism	mf	0.00E+00	3.92E-02	2.76E-02	0.00E+00	0.00E+00
	14	plasma membrane ATP synthesis coupled proton transport	bp	0.00E+00	3.85E-02	2.72E-02	0.00E+00	0.00E+00
	15	proton-transporting ATP synthase complex, coupling factor F(o)	cc	0.00E+00	8.44E-03	3.27E-03	0.00E+00	0.00E+00
	16	DNA-templated transcription, termination	bp	0.00E+00	8.98E-03	4.19E-03	0.00E+00	0.00E+00
	17	rRNA binding	mf	6.03E-02	1.47E-01	6.92E-02	1.20E-01	0.00E+00
	18	intracellular	cc	1.37E-01	2.30E-01	1.24E-01	1.88E-01	0.00E+00
1	19	glycolytic process	bp	4.82E-04	5.55E-03	0.00E+00	0.00E+00	0.00E+00
6 - amino acid synthesis & energy conversion	20	transferase activity, transferring acyl groups	mf	0.00E+00	0.00E+00	0.00E+00	5.26E-03	1.09E-02
	21	glutamine family amino acid biosynthetic process	bp	0.00E+00	0.00E+00	0.00E+00	8.98E-03	1.81E-02
	22	coenzyme binding	mf	0.00E+00	0.00E+00	0.00E+00	1.13E-02	2.16E-02
	23	ligase activity, forming carbon-nitrogen bonds	mf	0.00E+00	0.00E+00	0.00E+00	1.11E-02	2.11E-02
	24	ligase activity, forming carbon-sulfur bonds	mf	0.00E+00	0.00E+00	0.00E+00	3.08E-03	7.74E-03
	25	dicarboxylic acid metabolic process	bp	0.00E+00	0.00E+00	0.00E+00	4.49E-03	1.09E-02
	26	valine biosynthetic process	bp	0.00E+00	0.00E+00	0.00E+00	2.64E-03	8.46E-03
	27	isoleucine biosynthetic process	bp	0.00E+00	0.00E+00	0.00E+00	3.12E-03	9.44E-03
	28	oxidoreductase activity, acting on the CH-NH2 group of donors	mf	0.00E+00	0.00E+00	0.00E+00	1.69E-03	5.84E-03
	29	serine family amino acid metabolic process	bp	0.00E+00	0.00E+00	0.00E+00	6.83E-04	3.82E-03
	30	ketol-acid reductoisomerase activity	mf	0.00E+00	1.79E-03	5.64E-03	2.19E-03	7.21E-03
	31	ATP-binding cassette (ABC) transporter complex	cc	0.00E+00	5.45E-03	2.01E-02	7.65E-03	2.24E-02
	32	4 iron, 4 sulfur cluster binding	mf	0.00E+00	4.33E-03	1.06E-02	4.47E-03	1.43E-02
3 - translation binding & carbohydrate energy conversion	33	membrane	cc	0.00E+00	0.00E+00	0.00E+00	2.88E-01	2.57E-01
	34	transport	bp	2.45E-01	1.91E-01	0.00E+00	0.00E+00	0.00E+00
	35	RNA binding	mf	0.00E+00	0.00E+00	0.00E+00	1.92E-01	1.28E-01
	36	nucleoside-triphosphatase activity	mf	7.62E-02	9.97E-02	0.00E+00	0.00E+00	0.00E+00
	37	protein binding	mf	6.79E-02	9.27E-02	0.00E+00	0.00E+00	0.00E+00
	38	translation factor activity, RNA binding	mf	3.27E-02	4.79E-02	0.00E+00	0.00E+00	0.00E+00
	39	ATP binding	mf	1.23E-01	1.49E-01	0.00E+00	0.00E+00	0.00E+00
	40	alpha-amino acid metabolic process	bp	9.32E-03	0.00E+00	0.00E+00	1.84E-02	0.00E+00
	41	receptor activity	mf	1.47E-01	8.65E-02	1.17E-01	1.34E-01	1.14E-01
	42	cell outer membrane	cc	1.04E-01	5.65E-02	0.00E+00	0.00E+00	0.00E+00
	43	lyase activity	mf	0.00E+00	9.60E-03	1.68E-02	0.00E+00	0.00E+00
	44	monocarboxylic acid metabolic process	bp	0.00E+00	1.14E-02	1.88E-02	0.00E+00	0.00E+00
	45	metal ion binding	mf	0.00E+00	5.03E-02	7.45E-02	0.00E+00	0.00E+00
	46	oxidoreductase activity, acting on the aldehyde or oxo group of donors	mf	0.00E+00	3.95E-03	8.99E-03	0.00E+00	0.00E+00
	47	tricarboxylic acid cycle	bp	0.00E+00	7.03E-03	1.45E-02	8.01E-03	1.69E-02
	48	oxidation-reduction process	bp	0.00E+00	4.10E-02	6.76E-02	4.29E-02	8.45E-02
4 - nitrogen reallocation & vitamin B synthesis	49	glutamate-ammonia ligase activity	mf	0.00E+00	4.89E-03	1.74E-02	0.00E+00	0.00E+00
	50	pyridoxal phosphate binding	mf	0.00E+00	1.41E-03	4.99E-03	0.00E+00	0.00E+00
	51	glutamine biosynthetic process	bp	0.00E+00	4.72E-03	1.70E-02	0.00E+00	0.00E+00
	52	enzyme regulator activity	mf	0.00E+00	1.51E-03	5.97E-03	0.00E+00	0.00E+00
	53	nitrogen compound transport	bp	0.00E+00	1.48E-03	5.88E-03	0.00E+00	0.00E+00
	54	nitrogen fixation	bp	0.00E+00	3.98E-03	1.20E-02	0.00E+00	0.00E+00
	55	formate-tetrahydrofolate ligase activity	mf	0.00E+00	5.64E-04	3.27E-03	0.00E+00	0.00E+00
	56	folic acid-containing compound biosynthetic process	bp	0.00E+00	5.55E-04	3.30E-03	0.00E+00	0.00E+00
	57	tetrahydrofolate metabolic process	bp	0.00E+00	9.25E-04	4.27E-03	0.00E+00	0.00E+00
	58	glutamate synthase (NADPH) activity	mf	0.00E+00	2.82E-04	2.94E-03	0.00E+00	0.00E+00
	59	regulation of nitrogen utilization	bp	0.00E+00	5.55E-04	5.96E-03	0.00E+00	0.00E+00
	60	glutamate biosynthetic process	bp	0.00E+00	5.55E-04	5.00E-03	0.00E+00	0.00E+00
	61	thiamine biosynthetic process	bp	0.00E+00	1.85E-04	2.50E-03	0.00E+00	0.00E+00
5 - energy conversion & carbohydrate metabolism	62	oxidoreductase activity, acting on NAD(P)H, quinone or similar compound as acceptor	mf	6.61E-04	0.00E+00	0.00E+00	4.97E-03	0.00E+00
	63	nicotinamide nucleotide metabolic process	bp	6.43E-04	0.00E+00	0.00E+00	4.78E-03	0.00E+00
	64	nucleoside diphosphate phosphorylation	bp	9.64E-04	0.00E+00	0.00E+00	5.47E-03	0.00E+00
	65	metal ion transport	bp	3.21E-04	0.00E+00	0.00E+00	4.39E-03	0.00E+00
	66	single-organism catabolic process	bp	1.12E-03	6.39E-03	1.49E-02	5.76E-03	0.00E+00
	67	single-organism carbohydrate metabolic process	bp	1.45E-03	7.13E-03	1.39E-02	6.54E-03	0.00E+00
7 - formate & viral activity	68	molybdenum ion binding	mf	7.27E-03	1.69E-03	0.00E+00	2.19E-03	9.41E-03
	69	formate dehydrogenase (NAD+) activity	mf	6.45E-03	1.51E-03	0.00E+00	1.39E-03	6.68E-03
	70	outer membrane-bounded periplasmic space	cc	2.51E-02	9.16E-03	1.70E-02	1.31E-02	2.98E-02
	71	viral capsid	cc	4.48E-03	7.20E-04	0.00E+00	7.65E-04	0.00E+00
